# Oriented Multivalent Display Drives Consistent Serum Immunodominance to the Ebola Virus Glycoprotein

**DOI:** 10.1021/acscentsci.5c01886

**Published:** 2026-01-09

**Authors:** Chu Zheng, Adonis A. Rubio, Sheena Vasquez, Dominic Pham, Zhuangyu Pan, Christopher O. Barnes, Peter S. Kim

**Affiliations:** † Department of Biochemistry, Stanford University School of Medicine, Stanford, California 94305, United States; ‡ Sarafan ChEM-H, 6429 Stanford University, Stanford, California 94305, United States; § Department of Biology, 6429 Stanford University, Stanford, California 94305, United States; ∥ Stanford Biosciences, Stanford University School of Medicine, Stanford, California 94305, United States; ⊥ Stanford Biophysics Program, Stanford University School of Medicine, Stanford, California 94305, United States; # Chan Zuckerberg Biohub, San Francisco, California 94158, United States

## Abstract

Despite the vast diversity of B cell repertoires, serum antibody responses during viral infection often focus on a limited set of epitopes—a phenomenon known as immunodominance. This inherent bias establishes a hierarchy of epitope responses, which often facilitates viral immune evasion and presents a major challenge for universal vaccine design. It remains unclear whether serum immunodominance is primarily driven by antigen-intrinsic properties or by the spatial constraints imposed by virion-bound antigen presentation. Here, using Ebola virus glycoprotein (GP) as a model system, we found that trimeric GP elicited varied epitope hierarchies between individual animals during primary immunization. In contrast, multivalent GP presentation on either a vesicular stomatitis virus or ferritin nanoparticles—in the native orientation found on the Ebola virus—elicited highly consistent and more refined epitope hierarchies across multiple mice and guinea pigs. These findings reveal a key role of oriented multivalent presentation in shaping serum immunodominance. The striking consistency of epitope hierarchy among individuals suggests that oriented multivalent presentation may promote more uniform immune protection at the population level, beyond increasing the magnitude of antibody binding and neutralizing responses.

## Graphical abstract

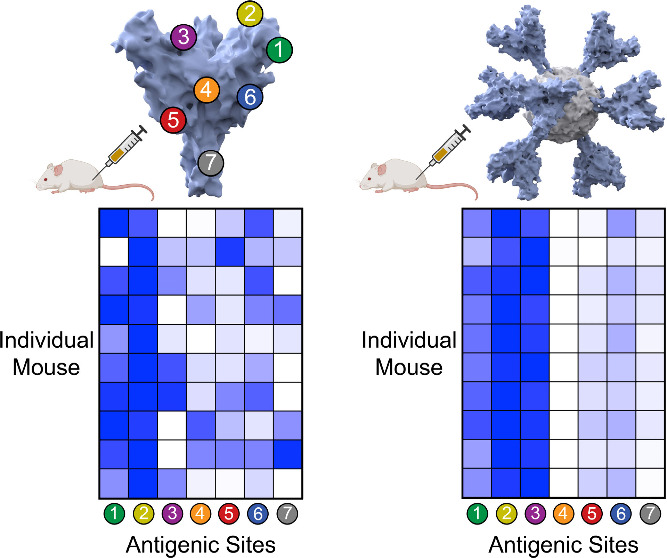

## Introduction

B cells generate a highly diverse antibody repertoire through stochastic processes such as V­(D)­J recombination and somatic hypermutation, enabling recognition of virtually any antigen. Despite this diversity, antibody responses often converge on specific regions of the antigen,^1–6^ a phenomenon known as antibody immunodominance. A well-known example is influenza A virus hemagglutinin (HA),^2,7–12^ which consistently elicits stronger responses to its head domain over the stem during infection and vaccination. The mechanistic basis of how such nonrandom patterns emerge from the inherent stochasticity of B cell activation and selection remains poorly understood.

A highly ordered multivalent arrangement of surface proteins is a hallmark of many viruses.^13–15^ Modern vaccine designs mimic this repetitive macromolecular presentation to enhance antibody binding and neutralizing responses.^16–18^ However, how multivalent antigen display shapes immunodominance remains unclear, and has only been discussed in a few antigen systems^13,14^ —often indirectly, and with mixed findings.^1,8,13,14,19,20^ Most studies have focused on responses to natural infection^9,12,21–23^ or inactivated virus vaccines,^7,9^ where antigens are inherently multimerized. Whether immunodominance is primarily driven by structural features of the antigen itself or by spatial constraints such as antigen orientation and density on the viral surface remains an open question. To address this, we used the Ebola virus (EBOV) glycoprotein (GP) as a model antigen to dissect the contributions of intrinsic antigen structure and oriented multivalent display.

As the sole protein on EBOV virus particles and the primary target of protective antibodies, GP is the primary antigen used for vaccine design.^24,25^ The full-length GP contains a mucin-like domain (MLD),^24–26^ which shields key antigenic regions from immune recognition and is cleaved during viral entry (Figure 1A). Due to its intrinsic disorder and dense glycosylation, most structural and epitope mapping studies use a truncated version of GP lacking the MLD (GPΔM).^24,25,27^ Here we focus on seven epitope classes on GPΔM defined by GP-specific monoclonal antibodies (mAbs)^24,25,27–32^ (Figure 1B). Six of the seven epitopes are targeted by neutralizing antibodies with subnanomolar potency^29,32–35^ (IC_50_ ∼ 0.1–1 nM; Table S1), except for epitope 2, where c13C6 is non-neutralizing.^25^ These epitopes cannot be characterized using linear epitope mapping approaches^36–39^ due to their discontinuous nature on the protein surface (Figure 1C, Figure S1), and their immunodominance patterns in serum responses remain unknown.

**Figure 1:**
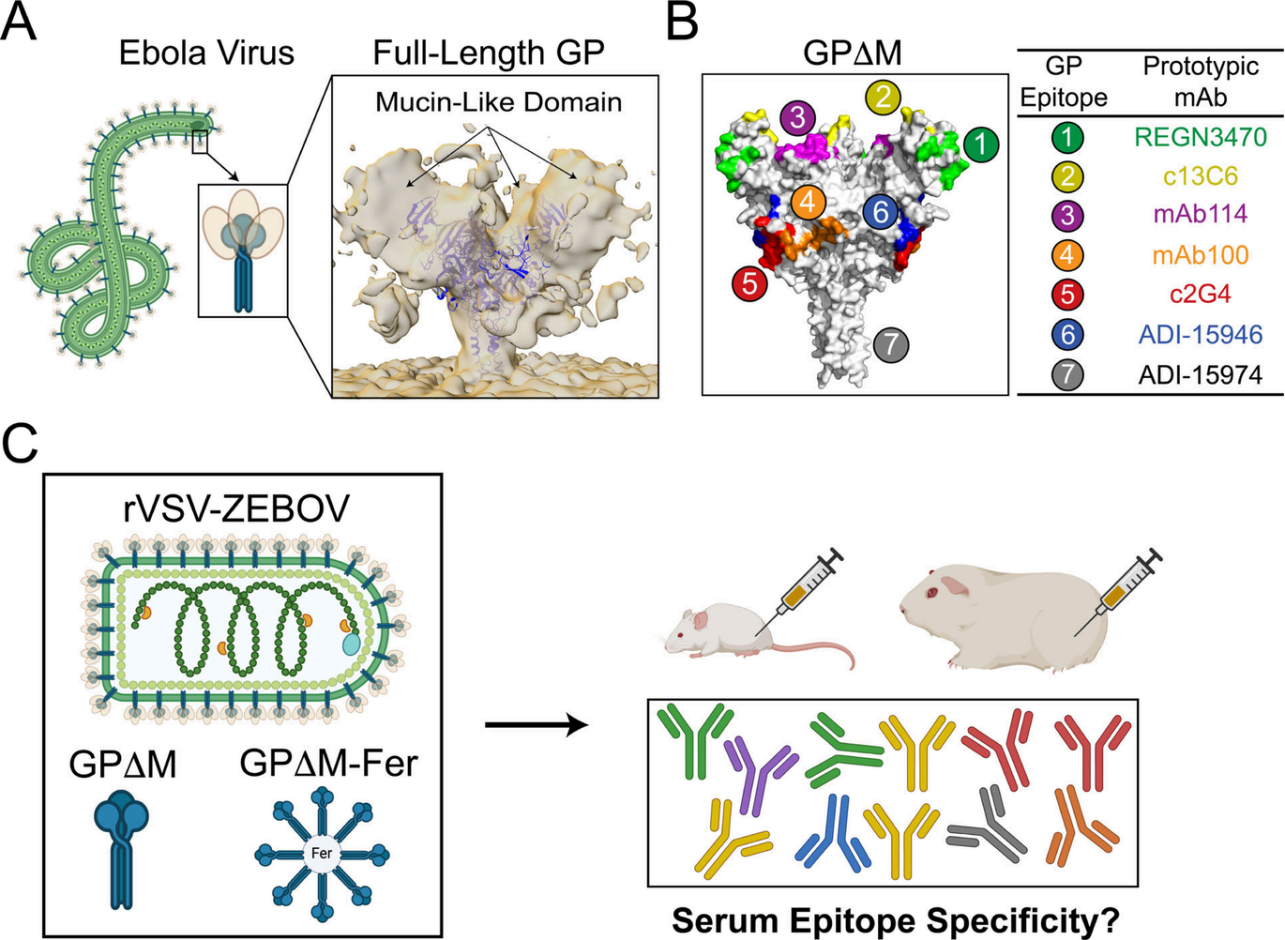
Profiling epitope hierarchy of serum antibody responses to the Ebola virus glycoprotein (GP). (A) Schematic of the full-length GP trimer on the Ebola virus surface. Overlay (blue ribbons) of the crystal structure of GPΔM, which lacks the (tan) mucin-like domain (PDB: 5JQ3) with the cryo-EM density map of full-length GP on the viral envelope (1.2σ contour; EMD-8630). (B) Crystal structure of GPΔM (PDB: 5JQ3), showing seven antigenic sites and their corresponding prototypic monoclonal antibodies used for blocking. Sequences of the seven epitopes in the context of the full-length GP are shown in Figure S1. (C) Schematic overview of serum antibody responses to GP elicited by different immunogen formats (the approved vaccine rVSV-ZEBOV, the GPΔM trimer, and GPΔM on ferritin nanoparticles, GPΔM-Fer) in mice and guinea pigs. Serum antibodies are color-coded based on their epitope specificities.

To assess how the structural context of antigen display dictates immunodominance, we compared serum epitope recognition across different immunization platforms—with and without oriented multivalent display—in both mice and guinea pigs (Figure 1C). To profile serum epitope hierarchy, we quantified the relative magnitude of serum responses by measuring titer fold-changes after specifically blocking each of the seven individual epitopes with monoclonal antibodies (mAbs). Our results show that oriented multivalent display of GP on vesicular stomatitis virus (VSV) or ferritin nanoparticles elicits consistent serum responses with a highly directed immunodominance hierarchy across both mice and guinea pigs. In contrast, initial immunization with trimeric GP produced more heterogeneous epitope profiles, which ultimately became more focused with boosting. These findings suggest that oriented multivalent display mirroring the native orientation found on the Ebola virus biases vaccine responses toward a more robust and directed immunodominance response among individuals.

## Results

### Epitope Hierarchy of Serum Responses to rVSV-ZEBOV in BALB/c

The rVSV-ZEBOV vaccine—currently the only FDA-approved EBOV vaccine^40^—uses vesicular stomatitis virus to express multiple copies of full-length GP on its surface (Figure 2A), mimicking natural infection. We immunized BALB/c mice and monitored anti-GP IgG titers, and also performed serum neutralization assays using a pseudovirus that expresses full-length GP on the viral surface. A single vaccine dose elicited anti-GP IgG titers of ∼ 10^3^ at week 3 (Figure 2B, Figure S2), which remained stable in subsequent weeks. Neutralizing titers (NT_50_) were mostly undetectable at week 3, but by week 7, six of ten mice showed measurable activity (geometric mean NT_50_ = 110; Figure S3), consistent with previous reports.^41^

**Figure 2:**
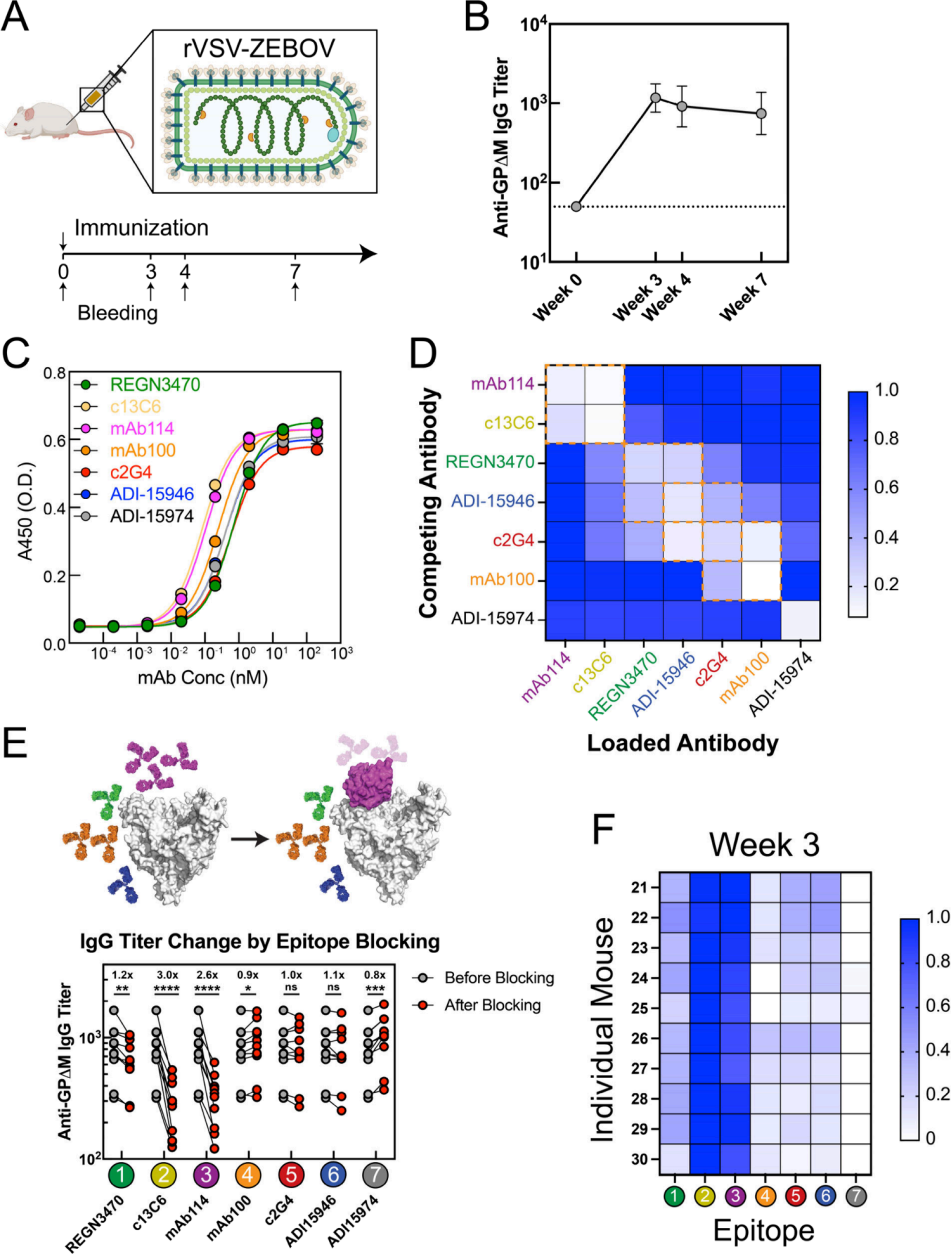
Serum antibody responses to rVSV-ZEBOV in mice. (A) BALB/c mice were immunized intramuscularly with a single dose of rVSV-ZEBOV (1 × 10^5^ PFUs). (B) Anti-GPΔM IgG titers measured across multiple time points postvaccination. (*n* = 10, error bars represent ± SD of log-transformed IgG titers). Data for individual animals are shown in Figure S2C. Dashed line indicates the limit of quantification. (C) ELISA curves of monoclonal antibodies binding to GPΔM. (D) Heatmap of pairwise competition among monoclonal antibodies measured by biolayer interferometry (BLI). The extent of competition is quantified by the reduction in BLI binding response of the test mAb after loading the competing mAb (SI Methods). White indicates strong competition; blue indicates weak or no competition. The orange dashed box highlights antibody pairs with high competition (>60%; see SI Methods). (E) Epitope-specific contributions to polyclonal responses quantified by competition ELISA. Top: schematic of the assay in which epitope-specific mAbs block defined antigenic sites on GP. Bottom: IgG titers before and after epitope blocking. Geometric mean titer fold-changes are shown above each column. Statistical significance was assessed using paired *t* tests on log-transformed titers. (F) Heatmap of epitope hierarchy at week 3. Each row represents an individual animal, with animal IDs labeled on the left; columns correspond to the seven GP epitopes. Color intensity reflects log-transformed titer fold-change values on a per-animal basis normalized by linearly scaling them from zero to one, with blue indicating stronger responses (unnormalized data are shown in Figure S6G).

Next, we quantified serum responses to the seven defined GP epitopes by using competition ELISA. Specifically, we measured reductions in IgG binding titers (half-maximal effective dilution, ED_50_) after epitope blocking with each specific mAb. All seven mAbs bound to GPΔM with EC_50_ = 0.1–1 nM (Figure 2C) and showed expected competition profiles in biolayer interferometry (Figure 2D), consistent with their known structural footprints.^28–32^ In control experiments, these mAbs remained fully bound to GP during the time of assay (Figure S4), confirming persistent epitope blocking. Because the ED_50_ reflects the overall strength of the polyclonal antibody response to GP, larger reductions in ED_50_ upon epitope blocking indicate stronger contributions from serum antibodies targeting that epitope. To quantify these contributions, we computed log-transformed titer fold-changes for each epitope (Figure 2E, Figure S5, Figure S6). Because both the preblocking and postblocking binding titers reflect contributions from antibodies targeting multiple epitopes, the resulting titer fold-changes may depend on the specific composition of each serum sample and thus are most meaningful when comparing epitope responses within the same sample. To focus on the relative distribution of epitope responses, which defines immunodominance hierarchy, we normalized these log-transformed titer fold-changes on a per-animal basis by linearly scaling these values from zero to one, enabling direct comparison across all samples (Figure 2F). To provide complementary views of the data, we also include the log titer fold-changes (Figure S5) and the unnormalized epitope hierarchy profiles (Figure S6).

At week 3 postvaccination, rVSV-ZEBOV elicited a highly consistent epitope recognition profile across all mice, showing a tight immunodominant serum response to epitopes 2 and 3 (Figure 2F). As a complementary metric, we analyzed changes in absorbance at 450 nm (ΔA450) after epitope blocking at the ED_50_ dilution, which yielded results consistent with the fold-change analysis—with epitopes 2 and 3 eliciting the dominant responses (Figure S7). Together, these data reveal a robust epitope hierarchy when full-length GP is multivalently displayed on the virion surface in the same orientation as on the native Ebola virus.

### Antigen-Intrinsic and Oriented Multivalent Display-Induced Epitope Hierarchies

To determine if oriented multivalent display such as that on rVSV-ZEBOV is essential for eliciting a clear consistent GP immunodominance profile, we next immunized mice with either recombinant GPΔM trimer or a ferritin nanoparticle displaying eight GPΔM trimers (GPΔM-Fer; Figure 3A),^42–45^ with boosts at weeks 3 and 6. We found that both immunogens elicited anti-GP serum responses in mice at week 3 (Figure 3B, Figure S2). Notably, GPΔM-Fer elicited ∼14-fold higher titers than GPΔM at week 3, despite both vaccinations containing similar total amounts of GPΔM trimer. This enhancement demonstrates the superior immunogenicity conferred by ferritin nanoparticle display, consistent with previous studies.^44^ GPΔM-Fer also outperformed GPΔM in neutralization. The GPΔM-Fer group showed an NT_50_ of ∼340 at week 3, whereas the GPΔM group’s NT_50_ was below 50 (Figure 3C, Figure S3). Following a booster vaccination, GPΔM-Fer NT_50_ increased to ∼2 × 10^4^, 55-fold higher than GPΔM (NT_50_ = 370). This difference narrowed after a second boost, with both groups converging at ∼10^4^.

**Figure 3:**
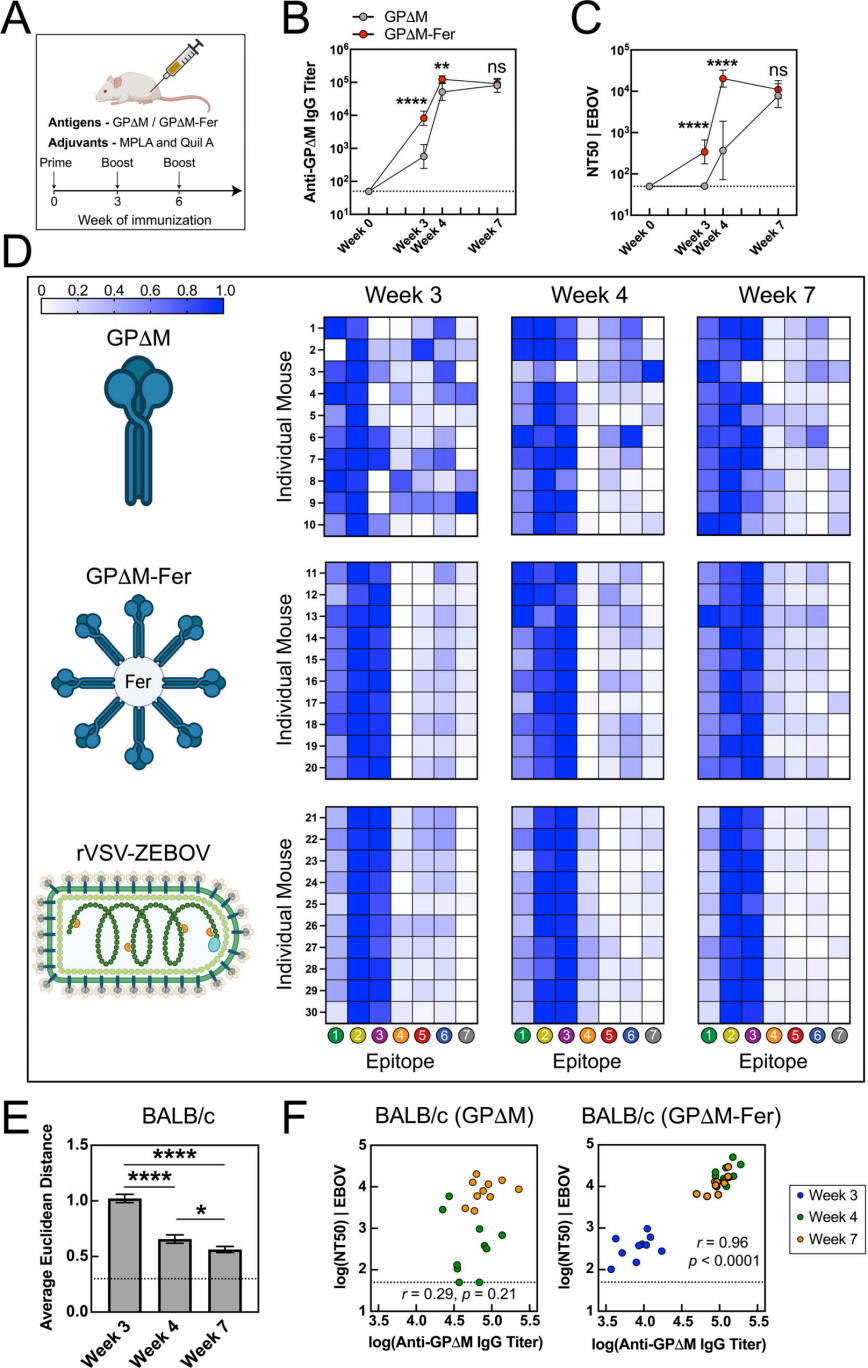
Serum antibody responses to GPΔM and GPΔM-Fer in mice. (A) BALB/c mice (*n* = 10 per group) were immunized intramuscularly with 5 μg of antigen adjuvanted with MPLA/Quil-A (10 μg/10 μg) on days 0, 21, and 42. (B,C) Anti-GPΔM IgG titers (B) and neutralization titers (C) elicited by GPΔM (gray) and GPΔM-Fer (red) in BALB/c mice. Data for individual animals are shown in Figure S1 and S2. In (B) and (C), data are presented as mean ± SD of log-transformed values. Statistical significance between vaccination groups was assessed by Welch’s *t* tests with Holm-Bonferroni correction. (D) Heatmaps of serum epitope hierarchy determined by competition ELISA in BALB/c mice at weeks 3, 4, and 7. Each row represents an individual animal, with animal IDs labeled on the left; columns correspond to the seven GP epitopes. Color intensity reflects log-transformed titer fold-change values after epitope blocking, with blue indicating stronger responses. (E) Similarity between GPΔM and GPΔM-Fer epitope hierarchy profiles in mice, quantified by the average pairwise Euclidean distance between GPΔM samples at each time point and the GPΔM-Fer group at week 7 (used as a reference). Dashed lines indicate the average within-group distance of the GPΔM-Fer group at week 7, serving as a baseline for comparison. Error bars represent the standard error; statistical significance was determined by Welch’s *t* tests with Holm-Bonferroni correction. (F) Correlation between IgG titers (ED_50_) and neutralization titers (NT_50_) in BALB/c mice with Pearson correlation coefficient (*r*) and *p*-value. Colors denote time points: blue (week 3), green (week 4), and orange (week 7).

Next, we compared the serum epitope hierarchy elicited by GPΔM and GPΔM-Fer by competition ELISA. Strikingly, both immunogens produced epitope profiles that persisted between weeks 3, 4, and 7, but with distinct epitope hierarchies. Specifically, GPΔM elicited a broad and variable response across multiple epitopes during the primary response, indicating the lack of a defined intrinsic epitope hierarchy (Figure 3D). In contrast, GPΔM-Fer elicited a highly consistent and focused response toward epitopes 1–3, closely mirroring the pattern induced by rVSV-ZEBOV (Figure 3D, Figure S8). Note that rVSV-ZEBOV, unlike GPΔM-Fer, presents a full-length GP containing the MLD on the viral surface. Given that GPΔM-Fer and rVSV-ZEBOV differ substantially in vaccine platform, the presence of the MLD, immunogenicity, and degree of multivalency—including both antigen density and the number of antigen copies per particle—yet share the same antigen orientation, their similar epitope hierarchy and response consistency suggest that antigen orientation is a key determinant of robust and consistent immunodominance patterns, whether on a viral surface or a nanoparticle scaffold. This is consistent with a prior study showing that antigen reorientation enhances immunofocusing and cross-reactivity.^46^

Although GPΔM immunization shows no consistent epitope hierarchy among individuals at week 3, boosting with the same immunogen led to a more focused serum response targeting epitopes 1–3 in mice (Figure 3D). As epitope recognition became more focused with GPΔM by week 7, it became similar to that shown by GPΔM-Fer throughout the course of the experiment. To quantify this convergence, we represented each sample’s epitope profile as a seven-dimensional vector and measured pairwise distances between GPΔM samples at each time point and the GPΔM-Fer group at week 7 as a reference (Figure 3E). The average distance decreased following each boost, indicating the emergence of a GP-intrinsic epitope hierarchy that, strikingly, matches the pattern shaped by ferritin display. The convergence of the epitope hierarchy after boosting raises the possibility that the lack of a clear hierarchy in the GPΔM group during primary immunization may partly result from its weaker immunogenicity compared to multimeric formats. However, the rVSV-ZEBOV group, despite having similarly low binding titers (Figure 2B), showed a consistent epitope hierarchy similar to the GPΔM-Fer group (Figure 3D), which had substantially higher binding titers (Figure 3B). This suggests that the variability observed in the GPΔM group is unlikely due to low binding titer. We also found that some residual binding remained after blocking mouse antisera with all seven reference mAbs (Figure S9), indicating that a portion of the serum response targets additional or partially overlapping epitopes. However, these responses do not affect the relative hierarchy among the seven defined epitopes. Since booster immunizations predominantly recall memory B cells generated during the primary response,^47^ these results suggest that the initial response established a memory repertoire inherently biased toward epitopes 1–3. This aligns with previous studies showing a prevalence of glycan cap-specific memory B cells in EBOV convalescent patients.^24,32,48^ Together, these findings demonstrate that multivalent display of GP, in its native orientation found on the Ebola virus, significantly amplifies the antigen-intrinsic epitope hierarchy.

Given the striking differences in epitope hierarchy between the GPΔM and GPΔM-Fer groups, we next asked whether epitope hierarchy is functionally relevant to serum neutralization. We found that the GPΔM-Fer group showed a strong positive correlation between ED_50_ and NT_50_ (*r* = 0.96, *p* < 0.0001), while there was no significant correlation in the GPΔM group (Figure 3F). We excluded week-3 data from GPΔM from the analysis because all NT_50_ values at this time point were below the limit of quantification. Although similarly removing week 3 data from GPΔM-Fer reduced the Pearson correlation from 0.96 to 0.71 (Table S2), it remained statistically significant (*p* = 0.0004) and was still much stronger than that of GPΔM, reinforcing the robustness of the difference. Furthermore, incorporating epitope hierarchy into a linear regression model along with ED_50_ improved correlation with NT_50_ for the GPΔM group in the mice (Figure S10), although this result requires validation in larger data sets beyond the scope of this work. Overall, these findings show that the consistent epitope hierarchy promoted by oriented multivalent display is associated with a strong correlation between ED_50_ and NT_50_, suggesting that epitope hierarchy may influence serum neutralization responses.

To evaluate the generality of these findings across species, we conducted parallel experiments in guinea pigs, an outbred animal model, using the GPΔM and GPΔM-Fer platforms and the rVSV-ZEBOV vaccine (Figure 4, Figures S11–S16). The effects of ferritin nanoparticle display on serum binding and neutralizing responses, as well as the resulting epitope recognition profiles (Figure 4D), closely recapitulate those observed in mice (Figure 3D), including the immunodominance of epitopes 1–3 for GPΔM-Fer and the convergence of the response on these epitopes for GPΔM. Interestingly, rVSV-ZEBOV immunized guinea pigs showed a more balanced serum response to epitope 1 relative to epitopes 2 and 3 compared to BALB/c mice, although both species showed immunodominant responses to epitopes 1–3. Together, these results further demonstrate the key role of oriented multivalent display in driving consistent serum immunodominance and suggest that the GP-intrinsic epitope hierarchy remains largely conserved across these animal models.

**Figure 4:**
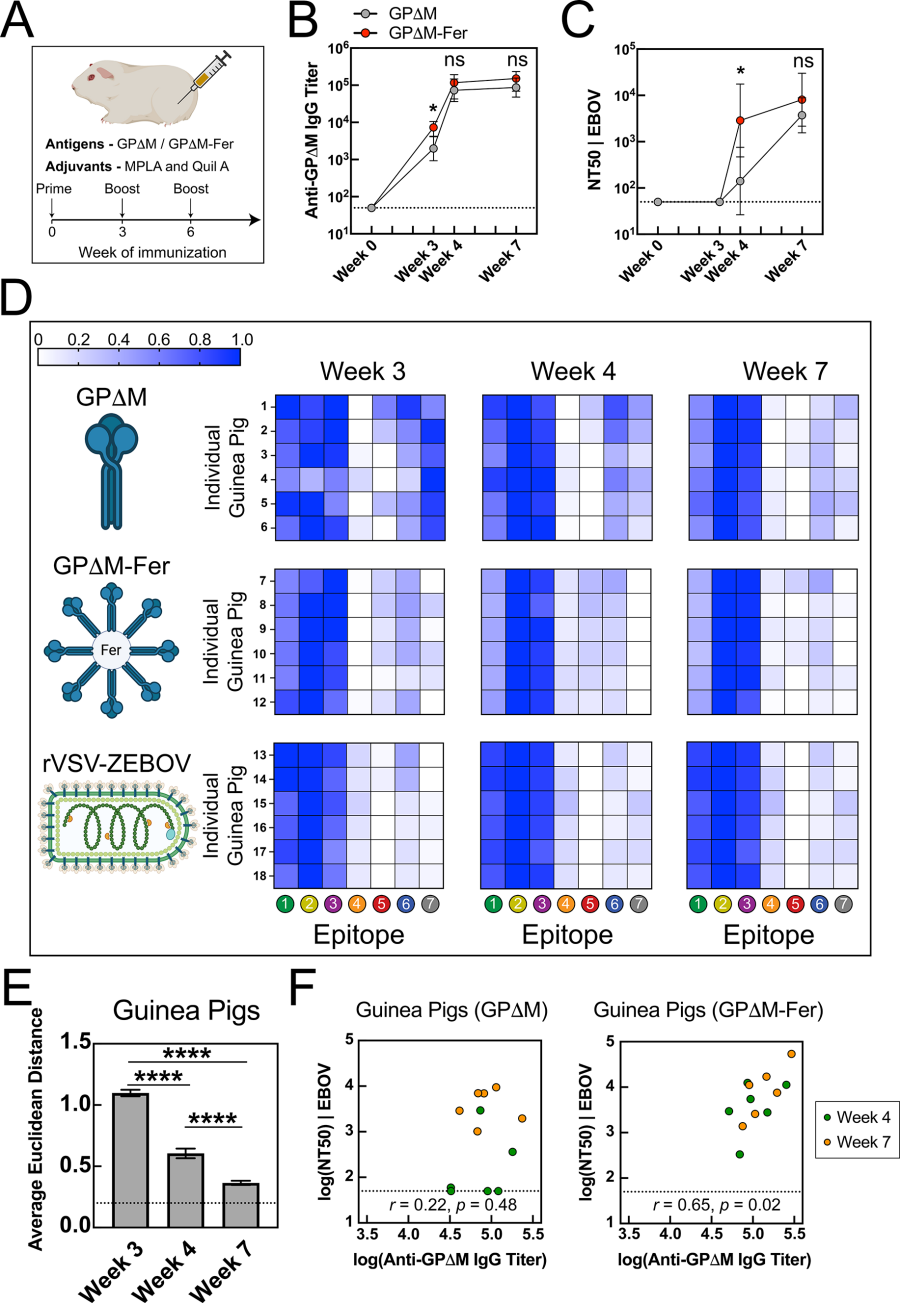
Serum antibody responses to GPΔM and GPΔM-Fer in guinea pigs. (A) guinea pigs (*n* = 6 per group) were immunized intramuscularly with 10 μg of antigen adjuvanted with MPLA/Quil-A (15 μg/15 μg) on days 0, 21, and 42. (B,C) Anti-GPΔM IgG titers (B) and neutralization titers (C) elicited by GPΔM (gray) and GPΔM-Fer (red) in guinea pigs. In (B) and (C), data are presented as mean ± SD of log-transformed values. Statistical significance between vaccination groups was assessed by Welch’s *t* tests with Holm-Bonferroni correction. (D) Heatmaps of serum epitope hierarchy in guinea pigs at weeks 3, 4, and 7. Each row represents an individual animal, with animal IDs labeled on the left; columns correspond to the seven GP epitopes. Color intensity reflects row-normalized, log-transformed titer fold-change values on a per-animal basis by linearly scaling them from zero to one, with blue indicating stronger responses. (E) Similarity between GPΔM and GPΔM-Fer epitope hierarchy profiles in guinea pigs, quantified by the average pairwise Euclidean distance between GPΔM samples at each time point and the GPΔM-Fer group at week 7 (used as a reference). Dashed lines indicate the average within-group distance of the GPΔM-Fer group at week 7, serving as a baseline for comparison. Error bars represent the standard error; statistical significance was determined by Welch’s *t* tests with Holm-Bonferroni correction. (F) Correlation between IgG titers (ED_50_) and neutralization titers (NT_50_) in guinea pigs with Pearson correlation coefficient (*r*) and *p*-value. Colors denote time points: green (week 4) and orange (week 7).

### Electron Microscopy-Based Polyclonal Epitope Mapping for EBOV GP

To directly visualize serum antibody binding to GPΔM, we performed negative-stain electron microscopy-based polyclonal epitope mapping (nsEMPEM) analysis of week 7 sera from guinea pig #3 in the GPΔM group and guinea pig #10 in the GPΔM-Fer group (Figure S17, Figure S18). Both animals exhibited strong IgG binding titers of ∼10^5^ (Figure 4B) and serum antibodies targeting the glycan cap (Figure 5). In the GPΔ-antiserum, nsEMPEM 3D reconstructions resolved a distinct class of stem-binding antibodies, whereas the GPΔM-Fer antiserum contained base-binding antibodies. Given that nsEMPEM preferentially detects abundant, high-affinity antibodies,^49^ these results suggest that glycan cap-targeting antibodies dominate the week 7 response in both groups. Supporting this, competition ELISA confirmed strong glycan cap responses and indicated that base- and stem-binding antibodies are likely present at lower abundance or affinity (Figure 4D). The detection of stem-binding antibodies by nsEMPEM, despite their small fold-changes in the competition ELISA, likely reflects both the limited sensitivity of the current competition ELISA format and partial rather than complete competition between ADI-15974 and the corresponding serum antibodies (Figure S17). This is further supported by our observation that mixing mouse antisera with 10 nM of the mouse IgG ADI-15974 results in only an ∼1.3-fold reduction in titer (Figure S19). Together, the two approaches provide complementary evidence for high-affinity serum antibodies targeting the glycan cap in the week 7 response across immunization groups, with additional epitope classes varying between GPΔM and GPΔM-Fer groups.

**Figure 5:**
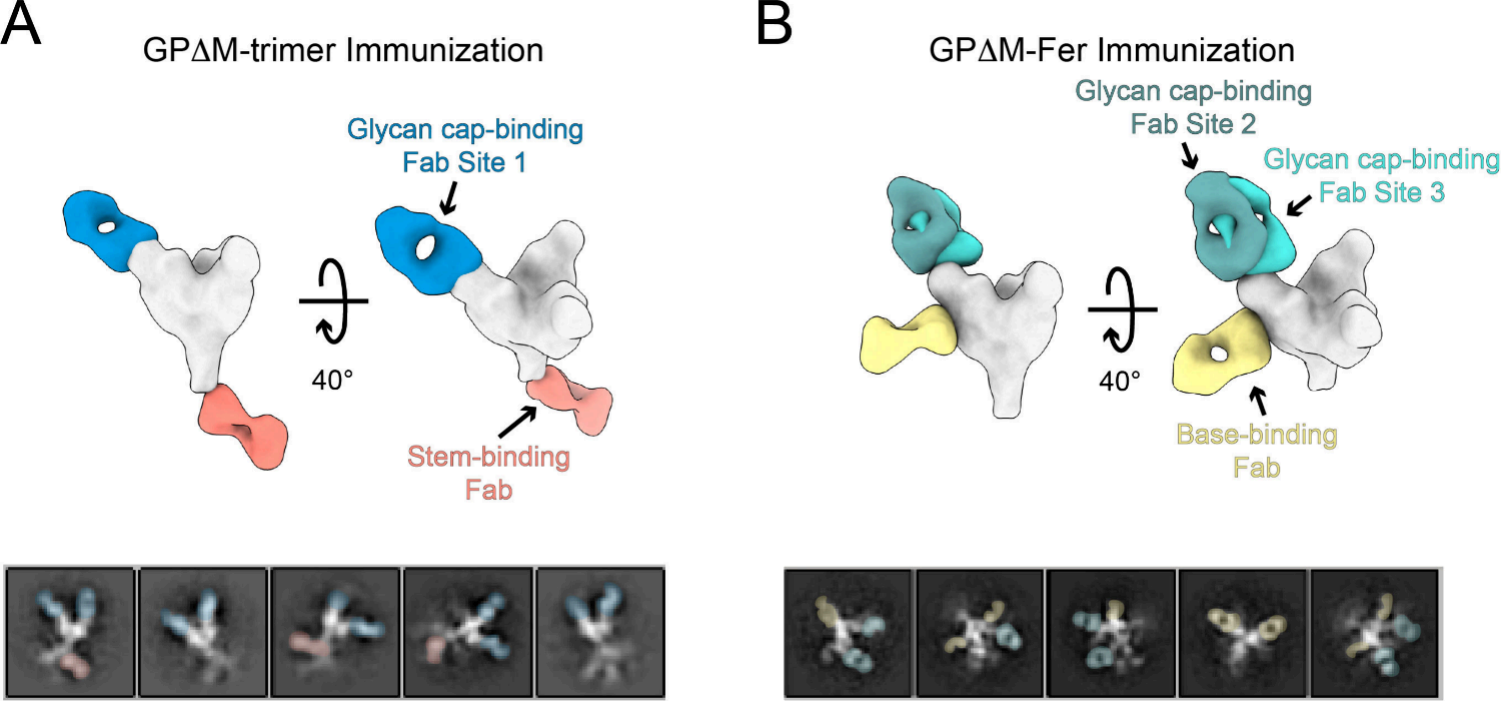
Negative-stain EMPEM of guinea pig antisera complexed with GPΔM trimer. (A) Composite map of GPΔM complexed with serum antibody Fabs from guinea pig #3 (immunized with GPΔM), with Fab densities modeled onto one GP protomer. Representative 2D classes are shown below. (B) Composite map of GPΔM complexed with serum antibody Fabs from guinea pig #10 (immunized with GPΔM-Fer), with Fab densities modeled onto one GP protomer. Representative 2D classes are shown below. Color scheme: Zaire Ebola GP (silver), Glycan cap-binding Fabs (light teal, dark teal, and blue), base-binding Fab (yellow), and stem-binding Fab (salmon).

## Discussion

Antibody immunodominance is widely recognized as a fundamental feature of B cell responses to viral infection and has been increasingly reported for emerging viruses,^21,23,50^ though often characterized only at the level of protein domains, and not more refined epitopes.^1,21,22,51^ Nearly every aspect of B cell biology—such as precursor frequency,^4,52–54^ B cell receptor affinity and avidity,^15,52^ and T cell help^55^—has been implicated in shaping immunodominance.^1,3^ However, the mechanistic dissection of these factors remains challenging due to the complexity of B cell responses and the lack of direct experimental data. In particular, it remains unclear whether serum immunodominance is primarily driven by intrinsic antigen structure or by its oriented, surface-bound presentation, as most previous studies have focused on natural infection or inactivated virus vaccination,^7,9,12,22,23,51,56^ where these two factors are inherently confounded. Here, we address this question by defining antigen-intrinsic hierarchy as the epitope pattern elicited by a soluble antigen and comparing it with responses to surface-bound antigens presented in a defined, native virus-like orientation. Using both virus- and protein-based display platforms in mice and guinea pigs, we demonstrate that oriented antigen presentation outweighs antigen-intrinsic hierarchy in eliciting consistent serum epitope hierarchy with a strong immunodominance pattern. These findings complement prior studies^14,15^ showing that multivalency increases B cell clonotype diversity and lowers the affinity threshold for B cell activation.

The current understanding of immunodominance has largely been rooted in studies of influenza HA,^2,7–12^ where some evidence suggests that head-domain dominance is intrinsic to HA structure,^1,8,19,57^ while other findings suggest a role for antigen presentation.^13,20,44^ Using EBOV GP as a structurally distinct antigen, this work provides direct, epitope-level evidence that antigen-intrinsic and surface display-induced hierarchies are not mutually exclusive. Our results show that trimeric GP elicits a weak intrinsic epitope hierarchy, which is obscured by variability in the initial response of individual animals, and becoming apparent only after boosting. Strikingly, the intrinsic hierarchy induced by trimeric GP after boosting closely matched the hierarchy elicited by multimerized GP in a native virus-like orientation. This observation indicates that the orientation of multivalent GP presentation on the Ebola virus amplifies—rather than alters—the antigen-intrinsic epitope hierarchy. Together with the intrinsic dominance of the HA head domain observed with soluble HA and its enhanced accessibility when presented on the influenza virus surface,^13^ this relationship between intrinsic epitope hierarchy and oriented, virion-bound antigen presentation may represent a common feature of serum immunodominance across viral antigens. These findings motivate future investigations into how reoriented antigen presentation (e.g., ref 43) influences immunodominance over repeated immunizations—whether the intrinsic epitope hierarchy eventually overtakes the initial hierarchy imposed by oriented, multivalent antigen display.

Multivalent display has been widely used in vaccine design, primarily due to its well-documented ability to enhance the magnitude of antibody binding and neutralizing responses—an effect attributed to improved B cell receptor cross-linking and stronger B cell activation.^17^ Our results suggest that the benefits of multivalent display may extend beyond increasing response magnitude. We show that highly oriented antigen presentation also promotes consistency in epitope recognition profiles across individuals compared to soluble antigens. This consistency may be critical for achieving more uniform immune protection at the population level.

## Supplementary Material



## Data Availability

All data are available in the main text or the Supporting Information. Final 3D reconstructions from the nsEMPEM data have been deposited to the Electron Microscopy Data Bank under the accession codes EMD-72372 (Map 4), EMD-72373 (Map 1), EMD-72374 (Map 2), EMD-72345 (Map 3).

## Notes

Additional experimental details, materials, and methods, including cell lines, antigen and antibody cloning, protein expression and purification, competition BLI, animal immunization studies, ELISAs, pseudotyped lentivirus production and neutralization assays, nsEMPEM, and ethics statements for animal experiments; figures showing structural representations of epitopes, individual mouse and guinea pig serum antibody titers and neutralization curves, competition ELISA validation and heatmaps of epitope hierarchy, correlation analyses between binding and neutralization, and nsEMPEM micrographs, 2D class averages, and composite maps; tables listing neutralization potencies of reference antibodies and Pearson correlation coefficients (PDF)

## Abbreviations

BLI: biolayer interferometry
EBOV: Ebola virus
ED_50_: half-maximal effective dilution
ELISA: Enzyme-Linked Immunosorbent Assay
GP: glycoprotein
HA: hemagglutinin
mAbs: monoclonal antibodies
MLD: mucin-like domain
nsEMPEM: negative-stain electron microscopy-based polyclonal epitope mapping
NT_50_: neutralizing titers
VSV: vesicular stomatitis virus
