# Correction to “Oriented Multivalent Display Drives Consistent Serum Immunodominance to the Ebola Virus Glycoprotein”

**DOI:** 10.1021/acscentsci.6c00134

**Published:** 2026-02-03

**Authors:** Chu Zheng, Adonis A. Rubio, Sheena Vasquez, Dominic Pham, Zhuangyu Pan, Christopher O. Barnes, Peter S. Kim

In the Data Availability Statement, the Electron Microscopy Data Bank (EMDB) accession code for the structure of Map 3 was incorrectly listed as EMD-72345. The correct accession code is EMD-72375.

The authors apologize for this error.

