# Supplementary material for: Oriented Multivalent Display Drives Consistent Serum Immunodominance to the Ebola Virus Glycoprotein
Source: ACS Cent Sci. 2026 Jan 9;12(1):100–10. doi: 10.1021/acscentsci.5c01886 (PMC12856675; doi:10.1021/acscentsci.5c01886)
Supplement: Supplementary file 1 [file oc5c01886_si_001.pdf]

Supporting Information

Oriented multivalent display drives consistent serum immunodominance to the Ebola virus glycoprotein

Chu Zheng[1,2], Adonis A. Rubio[2,3,4,‡], Sheena Vasquez[2,3,‡], Dominic Pham[2,5], Zhuangyu Pan[1,2], Christopher O. Barnes[2,3,6], and Peter S. Kim[1,2,6,*]

Affiliations:
[1]Department of Biochemistry, Stanford University School of Medicine, Stanford, CA 94305, USA.
[2]Sarafan ChEM-H, Stanford University, Stanford, CA 94305, USA.
[3]Department of Biology, Stanford University, Stanford, CA 94305, USA.
[4]Stanford Biosciences, Stanford University School of Medicine, Stanford, CA 94305, USA.
[5]Stanford Biophysics Program, Stanford University School of Medicine, Stanford, CA 94305, USA.
[6]Chan Zuckerberg Biohub, San Francisco, CA 94158, USA.
[‡]A.A.R. and S.V. contributed equally to this work.
[*]Corresponding author. Email: kimpeter@stanford.edu

Materials and Methods

S1. Cell lines.

HEK-293T cells (American Type Culture Collection) were cultured in D10 medium—Dulbecco's Modified Eagle Medium (DMEM; ThermoFisher, Cat. 11995081) supplemented with 10% fetal bovine serum (GeminiBio) and 1% L-glutamine/penicillin/streptomycin (GeminiBio, Cat. 100-500). Expi-293F cells (ThermoFisher, Cat. A14527) were maintained in a 2:1 (v/v) mixture of Freestyle medium and Expi-293 medium (Thermofisher, Cat. 12338026 and Cat. A1435102) in polycarbonate shaking flasks (Triforest Labware). Stellar competent E. coli cells (Takara Bio, Cat. 636766) were used for cloning and plasmid preparation.

S2. Antigen and Antibody Cloning.

A DNA sequence encoding the ectodomain of Ebola GP (Mayinga, 1976) with the mucin-like domain deleted (GPΔM; residues 1-308 and 491-656) was cloned into the pADD2 mammalian expression vector using In-Fusion cloning (Takara Bio, Cat. 638944). The transmembrane region was replaced with a foldon trimerization domain, followed by an Avi-Tag and a hexahistidine tag. To generate GP-Fer, GPΔM was fused to H. pylori ferritin (residues 5-168) via a Ser-Gly-Gly

linker and inserted into the pADD2 vector. For antibody constructs, gene fragments encoding the variable regions of the heavy chain (HC) and light chain (LC) were cloned into plasmids containing either human IgG or mouse IgG2a constant regions by In-Fusion. All constructs were verified by Sanger sequencing (MCLAB). For transfection, plasmids were transformed into Stellar cells (Takara Bio), purified using Maxiprep kits (Macherey Nagel, Cat. 740414.100), sterile-filtered (0.22-µm) in a biosafety cabinet, and stored at −20 °C until use.

S3. Protein Expression and Purification.

All proteins were expressed in Expi-293F cells cultured at 37°C with constant shaking (120 rpm) in a humidified incubator containing 8% CO_2. Cells were transfected at a density of 3 – 4 × 10^6 cells/mL using FectoPRO transfection reagent (Polyplus). For a 100 mL transfection, 60 µg of plasmid DNA was diluted into 10 mL of medium (67% Freestyle and 33% Expi-293) and mixed with 130 µL of FectoPRO. The mixture was incubated at room temperature for 10 minutes before being added dropwise to the cell culture. After transfection, D-glucose (4 g/L; Sigma-Aldrich) and valproic acid (3 mM; Thermo Fisher, Cat. 271830250) were added to boost protein expression. For antibody production, an equal mass of heavy-chain and light-chain plasmids (30 µg each per 100 mL transfection) was used. Biotinylated antigens were expressed using the same protocol with an engineered Expi-293 cell line expressing BirA[1]. Cells were harvested 3 – 5 days post-transfection by centrifugation at 7,000 × g for 5 minutes. The supernatant was filtered through a 0.22 µm membrane before further purification.

His-tagged proteins were purified using HisPur Ni-NTA resin (Thermo Fisher, Cat. 88223). Filtered supernatants were incubated with Ni-NTA resin (1 mL resin per 200 mL supernatant), and imidazole was added to a final concentration of 10 mM. The mixture was gently stirred overnight at 4 °C and then applied to a gravity-flow column. Bound proteins were washed with 20 mM imidazole in HEPES buffer saline (HBS; 20 mM HEPES, pH 7.4, 150 mM NaCl), and eluted using 250 mM imidazole in HBS. Eluted proteins were concentrated with centrifugal filters (Amicon Ultra 50K Centrifugal Filters, Millipore Sigma, Cat. UFC905096), buffer-exchanged into HBS, and further purified by size-exclusion chromatography (SEC) using a Superose® 6 Increase 10/300 GL column (Cytiva). SEC peak fractions were pooled, concentrated, buffer-exchanged into HBS containing 10% glycerol, and sterile-filtered through a 0.22-µm membrane.

GP-Fer nanoparticles were purified by anion-exchange chromatography followed by SEC. Filtered supernatants were applied to a HiTrap Q HP column (Cytiva) using an ÄKTA purification system (Cytiva). After washing with 20 mM Tris buffer (pH 8.0), proteins were eluted using a linear NaCl gradient (0 – 1.0 M). Fractions containing nanoparticles were concentrated using Amicon Ultra 100K Centrifugal Filters (Millipore Sigma, Cat. UFC910096) and further purified by two SEC runs using an SRT SEC-1000 column (Sepax Technologies). Peak fractions were pooled, concentrated, buffer-exchanged into HBS with 10% glycerol, and filtered through a 0.22-µm membrane.

Antibodies were purified using MabSelect PrismA protein A chromatography. Filtered supernatants were applied directly to a MabSelect PrismA column (Cytiva) on an ÄKTA system. After washing with HBS, bound antibodies were eluted with 15 mL of 100 mM glycine (pH 2.8)

into tubes containing 2 mL of 1 M Tris buffer (pH 8.0) for immediate neutralization. Fractions were concentrated and buffer-exchanged to HBS with 10% glycerol.

Protein concentrations were determined by absorbance at 280 nm, and purity was assessed by SDS-PAGE. Final protein samples were flash-frozen in liquid nitrogen and stored at −20 °C.

S4. Competition Biolayer Interferometry (BLI)

Competition BLI was measured using an Octet RED96 system (ForteBio) in 96-well flat-bottom black plates (Greiner). All samples were prepared in PBS with 0.1% BSA and 0.02% Tween-20 and assays were performed under agitation at 1000 rpm. Biotinylated antigens (200 nM) were immobilized on streptavidin biosensors (Sartorius). Following antigen loading, biosensors were dipped into wells containing buffer alone for 20 s, then into wells containing the first antibody to allow binding. After loading the first antibody, biosensors were again dipped in buffer for 20 s before being transferred to wells containing the second antibody. The extent of competition imposed by the first antibody on the binding of the second antibody was quantified as: $\text{Competition} = 1 - \frac{[\text{binding response with the first mAb loaded}]}{[\text{binding response without the first mAb}]}$.

S5. Animal Immunization Studies

Female BALB/c mice (6–8 weeks old) were obtained from The Jackson Laboratory, and female Dunkin-Hartley guinea pigs (1–2 months old, 400–600 g) were purchased from Charles River Laboratories. Antigens were formulated with adjuvants before injection. The total injection volume was adjusted to 100 μL in DPBS for BLAB/c mice and 200 μL for guinea pigs. Blood samples were collected into serum gel tubes (Sarstedt, Cat. 41.1378.005), centrifuged at 10,000 ×g for 5 minutes, and the resulting sera were stored at −80 °C.

S6. ELISAs

To immobilize biotinylated antigen, streptavidin was first coated onto 96-well plates (Thermo Scientific Clear Flat-Bottom Immuno Nonsterile 96-Well Plates, Thermo Fisher Scienticfic, Cat. 456537) through hydrophobic interactions. Specifically, 60 μL of streptavidin (4 μg/mL in PBS) was added to each well and incubated for 1 hour at room temperature. Plates were washed with Milli-Q water, then blocked with 120 μL of ChonBlock (Chondrex, Inc., Cat. 90681) and incubated overnight at 4 °C. For all subsequent steps, samples and dilutions were prepared in PBS containing 0.1% BSA and 0.02% Tween-20. Between each incubation, plates were washed three times with 300 μL of PBST. Biotinylated antigen (2 μg/mL) was added at 50 μL per well and incubated for 1 hour. Serial dilutions of antibodies or antisera were then added and incubated for 1 hour. Monoclonal antibodies were diluted starting from 200 nM in 10-fold serial dilutions, while antisera were diluted starting at 1:50 in 5-fold serial dilutions. After primary incubation, 50 μL of HRP-conjugated secondary antibody was added and incubated for 40 minutes. The following secondary antibodies were used: goat anti-mouse IgG (1:4000; Southern Biotech, Cat. 1037-05), goat anti-human IgG (1:4000; Southern Biotech, Cat. 2014-05), or donkey anti-guinea pig IgG (1:5000; Jackson ImmunoResearch, Cat. 706-035-148). Plates were washed six times with PBST before incubation with 50 μL of TMB substrate (Thermo Scientific, Cat. 34022) for 5 minutes.

Reactions were stopped by adding 50 µL of 2 M sulfuric acid, and absorbance at 450 nm was measured using a Tecan M200 plate reader.

For competition ELISA, competing antibodies in the human IgG format (REGN3470, c13C6, mAb114, mAb100, c2G4, ADI-15946, or ADI-15974; each at 100 nM) were pre-incubated with the immobilized antigen for 1 hour prior to the addition of serially diluted antisera. To confirm that antisera do not displace the blocking antibody, the ELISA signal of each blocking antibody was measured before and after incubation with serially diluted antisera under the same conditions used for the competition ELISA (100 nM blocking antibody and 1-hour incubation with serially diluted antisera), and the results are shown in Figure S4 To assess epitope hierarchy, log-transformed titer fold change values from triplicate experiments (performed on different days) were averaged and then normalized to enable direct comparison across all samples.

S7. Production of EBOV GP-Pseudotyped Lentivirus

EBOV GP-pseudotyped lentiviral particles encoding a luciferase-ZsGreen reporter were generated in Expi-293F cells using a five-plasmid system, as described previously[2,3]. The plasmid mixture included a packaging vector (pHAGE-Luc2-IRES-ZsGreen), three helper plasmids (pHDM-Hgpm2, pHDM-Tat1b, and pRC-CMV_Rev1b), and a plasmid encoding full-length EBOV GP. Expi-293F cells were transfected at a density of $3 – 4 \times 10^6$ cells/mL using the BioT reagent. For a 100 mL transfection, 100 µg of packaging vector, 34 µg of the EBOV GP expression plasmid, and 22 µg of each helper plasmid were combined in 10 mL of medium (67% Freestyle and 33% Expi-293), mixed with 300 µL of BioT, and incubated at room temperature for 10 minutes. The transfection mixture was then added dropwise to the cell culture. Immediately following transfection, D-glucose (4 g/L; Sigma-Aldrich) and valproic acid (3 mM; Thermo Fisher, Cat. 271830250) were added to boost viral production. After three days, cells were pelleted by centrifugation, and the supernatant containing pseudovirus was collected, filtered through a 0.45-µm membrance and then aliquoted, flash frozen in liquid nitrogen, and stored at −80 °C.

S8. Serum Neutralization Assays Using Pseudoviruses

As previously described[2], HEK-293T cells were seeded at a density of 2×10^4 cells per well in white-walled 96-well plates (Fisher Scientific, Falcon 353296) one day prior to infection (day 0). On day 1, mouse antisera were heat-inactivated at 56 °C for 30 minutes, serially diluted in D10 medium, and incubated with EBOV GP-pseudovirus (diluted in D10 medium supplemented with polybrene at 1:1000, v/v) for 1 hour at 37 °C. The virus-serum mixtures were then added to the pre-seeded HEK-293T cells. On day 4, the culture medium was removed, and 80 µL of luciferase substrate (BriteLite Plus; Perkin Elmer, MIDSIC, Cat. 6066769) was added to each well. Luminescence was measured using a Tecan M200 microplate reader. Percent infection was calculated relative to control wells containing virus only (100% infection) and uninfected cells (0% infection). Neutralization titers (NT_{50}) were defined as the serum dilution resulting in 50% reduction of luminescent signal. All assays were performed in technical duplicates.

S9. Negative-Stain Electron Microscopy-based Polyclonal Epitope Mapping (nsEMPEM) Sample Preparation

Sample preparation was adapted from a previously described protocol[4]. In brief, polyclonal IgGs were purified from heat-inactivated guinea pig antisera (n = 1 per treatment arm) using HiTrap MabSelect SuRe columns (Cytiva) and eluted in ProA Elution buffer (100 mM sodium citrate, pH 3.0, 150 mM sodium chloride). The collected elution was neutralized with 2 M Trizma (Sigma-Aldrich, cat. no. T3069) to a final pH of 6-7. The neutralized IgG samples were then digested with 1.5% w/w papain (Sigma-Adrich, cat. no. P3125) in Digestion Buffer (25 mM sodium phosphate pH 7.0, 10 mM EDTA, 10 mM L-cysteine) at 37°C, 100 rpm for 1 hour. Free Fab was isolated from the digestion reaction using MabSelect SuRe columns and further purified in Tris-buffered saline (TBS; 20 mM Trizma, pH 8.0, 150 mM sodium chloride, 0.02% v/v sodium azide) following size-exclusion chromatography on a Superdex 200 Increase 10/300 GL column (Cytiva). Subsequently, EBOV GP trimers (20 μg) were complexed with polyclonal Fab at a 1:25 ratio (w/w) overnight at room temperature before purification via size-exclusion chromatography on a Superose 6 Increase 10/300 GL column (Cytiva) against TBS. Fractions containing GP:Fab immune complexes were pooled and deposited for 60 s at approximately 12 μg ml^{-1} on freshly glow-discharged, carbon film on 300 mesh, copper grids (Ted Pella, cat. no. 01843 for GP-Ferritin samples and Electron Microscopy Sciences, CF300-CU-50 for GP-Trimer samples). Grids were then washed three times with MilliQ water, followed by staining with uranyl formate (2% w/v).

S10. nsEMPEM Data Collection and Processing

Data were collected on a Glacios 2 Transmission Electron Microscope equipped with a Falcon 4i detector (Thermo Fisher). Images were acquired at 57,000x magnification (2.5 Å/pixel) using 1.5 μm under focus, 200 kV, and 20 el/$Å^2$ dose. All data were processed in Relion 5.0. After performing CTF estimations (CTFFIND-4.1), approximately 1200 particles were manually picked from a subset of 50 images and used for automatic particle picking in Topaz on the entire datasets. Using a cutoff of -3, particles were extracted at 128 pixels. For the GP-Ferritin sample, a total of 5,495,721 particles were further 2D classified to yield 3,364,369 particles. These particles were refined against an initial 60-Å low-pass filtered volume of GP (PDB 5KEL) prior to 3D classification and 3D refinement. Three final maps were obtained: Map 1 (476,331 particles), Map 2 (537,357 particles), and Map 3 (112,157 particles). For the GP-Trimer sample, a total of 4,167,914 particles were 2D classified to yield 3,478,855 particles. These particles were refined against an initial 60-Å low-pass filtered volume of GP (PDB 5KEL) prior to 3D classification and 3D refinement to obtain Map 4 (139,445 particles).

Composite map figures were made in ChimeraX. Briefly, Maps 1-4 were low-pass filtered to 20-Å and aligned onto a 20-Å low-pass filtered volume of GP (PDB 5JQ7). Fab volumes (PDB 6DZN, molmap 5-Å, Gaussian filter 5-Å) were then docked into the corresponding Fab densities within Maps 1-4.

S11. Ethics Statement

All animal procedures were conducted in compliance with the Public Health Service Policy for Humane Care and Use of Laboratory Animals, under protocols approved by the Stanford University Administrative Panel on Laboratory Animal Care (APLAC-33709 for mice and APLAC-33765 for guinea pigs).

Supplementary Figures

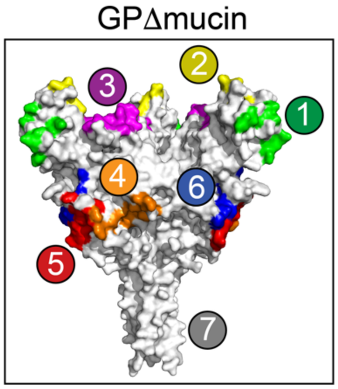

GP Epitope	Prototypic mAb
1	REGN3470
2	c13C6
3	mAb114
4	mAb100
5	c2G4
6	ADI-15946
7	ADI-15974

Signal Peptide

MGVTGILQLPRDRFKRTSFFLWVIILFQRTFSIPLGVIHNSTLQVSDVDKLVCRDKLSSTNQLRSVGL
NLEGNGVATDVPSATKRWGFRSGVPPKVVNYEAGEWAENCYNLEIKKPDGSECLPAAPDGIRGFP
RCRYVHKVSGTGPCAGDFAFHKEGAFFLYDRLASTVIYRGTTFAEGVVAFLILPQAKKDFFSSHPLRE
PVNATEDPSSGYYSTTIRYQATGFGTNETEYLFEVDNLTYVQLESRFTPQFLLQLNETIYTSGKRSNTT
GKLIWKVNPEIDTTIGEWAFWETKKNLTRKIRSEELSFTVVSNGAKNISGQSPARTSSDPGTNTTTED
HKIMASENSSAMVQVHSQGREAAVSHLTTLATISTSPQSLTTKPGPDNSTHNTPVYKLDISEATQVE Mucin-Like Domain
QHHRRTDNDSTASDTPSATTAAGPPKAENTNTSKSTDFLDPATTTSPQNHSETAGNNNTHHQDTG
EESASSGKLGLITNTIAGVAGLITGGRRTRREAIVNAQPKCNPNLHYWTTQDEGAAIGLAWIPYFGPA
AEGIYIEGLMHNQDGLICGLRQLANETTQALQLFLRATTELRTFSILNRKAIDFLLQRWGGTCHILGP
DCCIEPHDWTKNITDKIDQIIHDFVDKTLPDQGDNDNWWTGWRQWIPAGIGVTGVIIAVIALFCICKFVF

Transmembrane Domain

Figure S1. Structural representation and sequence mapping of the seven defined EBOV GP epitopes. Top: Crystal structure of GPΔM (PDB: 5JQ3) with the seven defined epitopes mapped onto the protein surface (left) and their corresponding mAbs (right) color coded by epitope. Bottom: Annotated amino acid sequence of full-length EBOV GP, with each epitope highlighted using the same color scheme as in the structural model. Bolded residues indicate amino acids shared by two or more epitope regions.

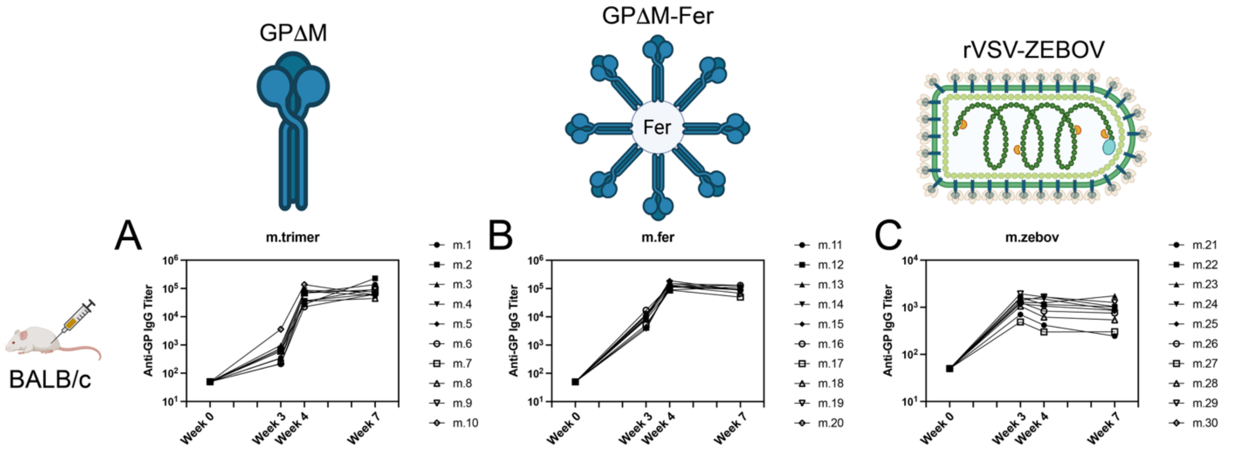

Figure S2. Individual mouse anti-GPΔM IgG titers across all immunization groups and timepoints. **A**, BALB/c mice immunized with GPΔM trimer. **B**, BALB/c mice immunized with GPΔM-Fer. **C**, BALB/c mice immunized with rVSV-ZEBOV.

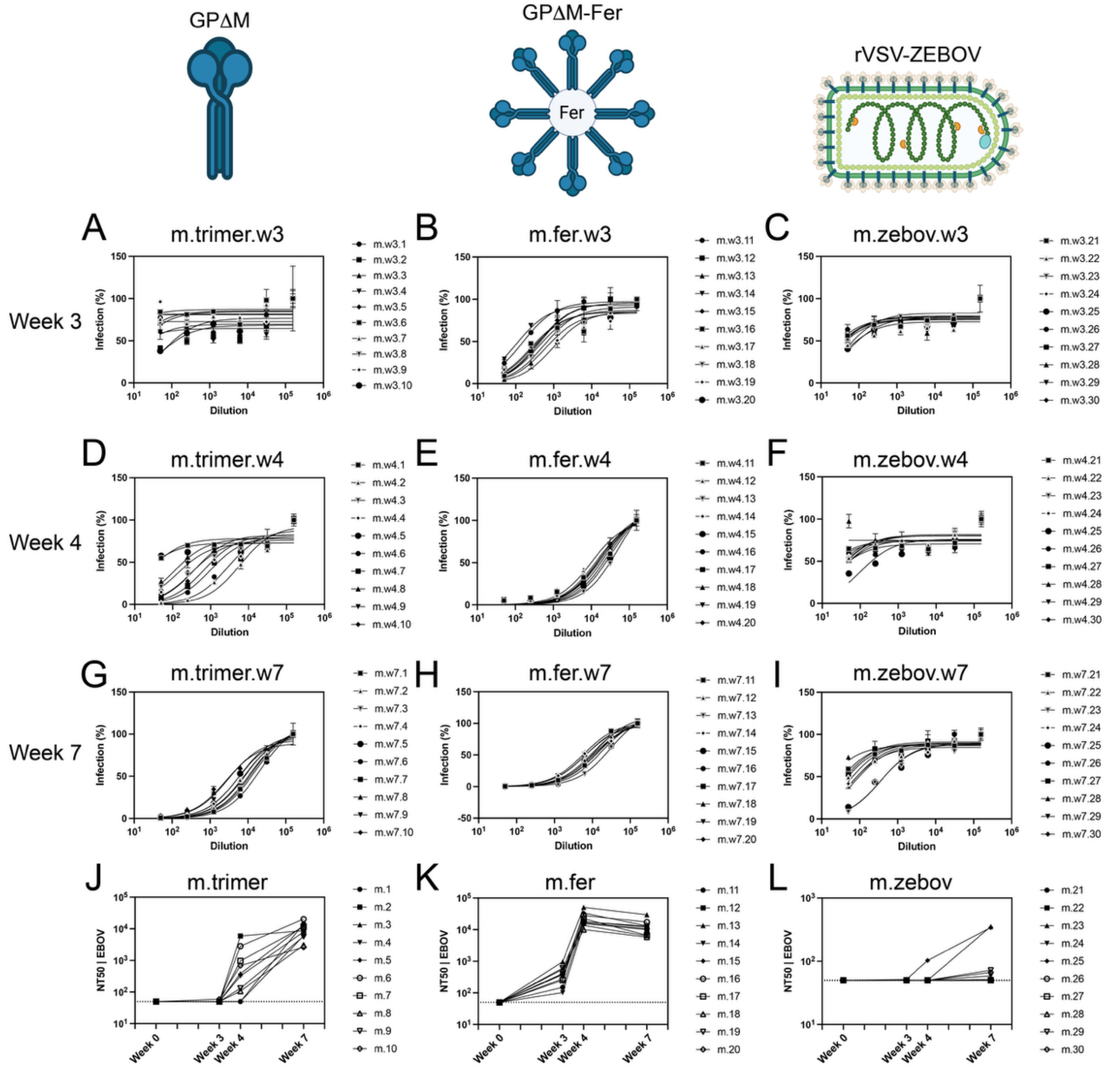

Figure S3. Pseudotyped virus neutralization titers for individual BALB/c mice across all immunization groups and timepoints. **A**-**C**, Neutralization curves at week 3 for the GPΔM (**A**), GPΔM-Fer (**B**), and rVSV-ZEBOV (**C**) groups. **D**-**F**, Neutralization curves at week 4 for the GPΔM (**D**), GPΔM-Fer (**E**), and rVSV-ZEBOV (**F**) groups. **G**-**I**, Neutralization curves at week 7 for the GPΔM (**G**), GPΔM-Fer (**H**), and rVSV-ZEBOV (**I**) groups. **J**-**L**, Summary plots of individual mouse neutralization titers (NT_{50}) across timepoints for the GPΔM (**J**), GPΔM-Fer (**K**), and rVSV-ZEBOV (**L**) groups. Dashed lines in **J**-**L** indicate the assay's lower limit of quantification.

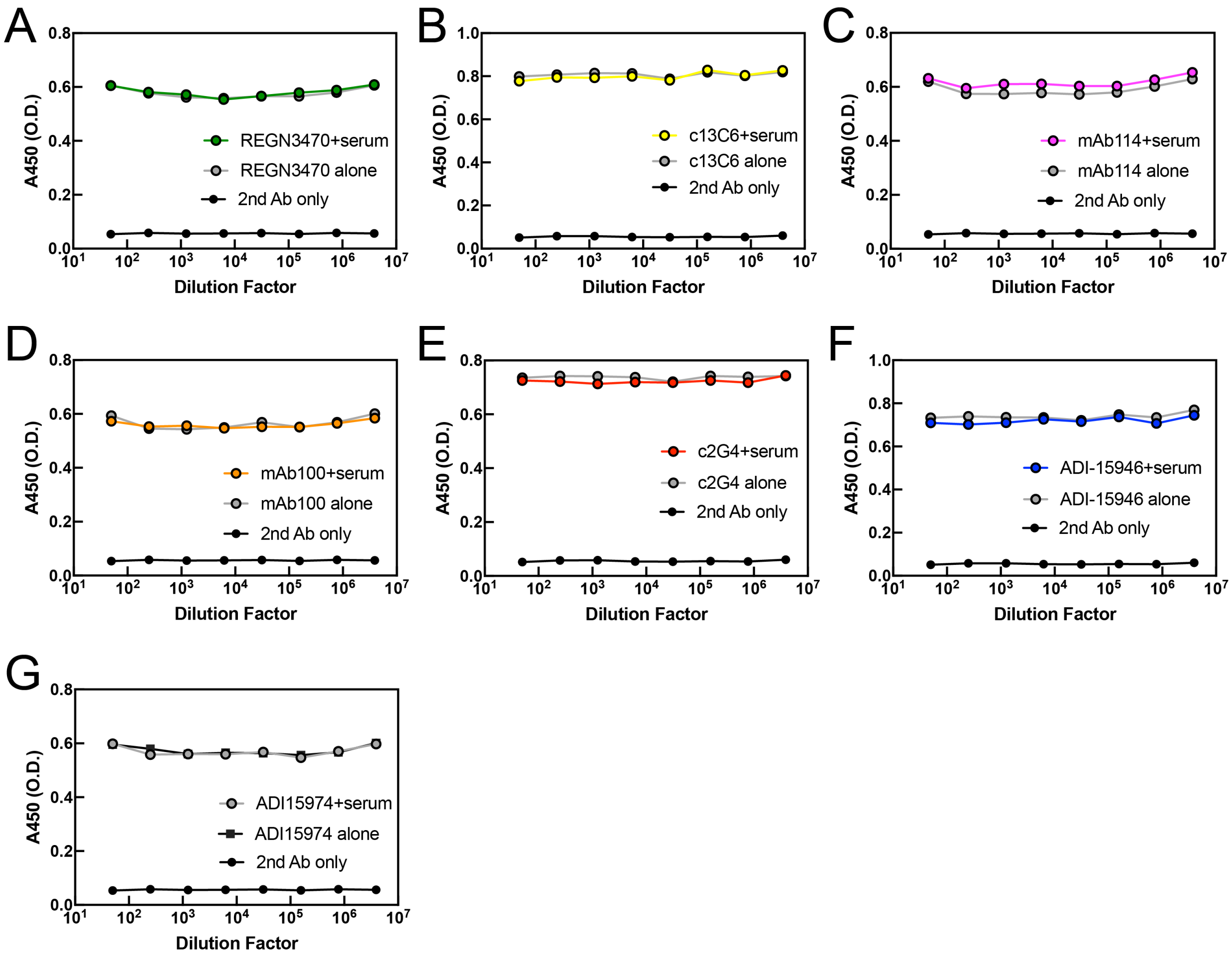

Figure S4. ELISA signal of monoclonal antibodies used for epitope blocking before and after incubation with serially diluted mouse antiserum. The antiserum used had an anti-GP titer of ~10^4. Panels show results for: REGN3470 (**A**), c13C6 (**B**), mAb114 (**C**), mAb100 (**D**), c2G4 (**E**), ADI-15946 (**F**), and ADI-15974 (**G**). Gray curves represent mAb binding in the buffer (PBS containing 0.1% BSA and 0.02% Tween-20) without incubation with antiserum. Colored curves show mAb binding following incubation with serially diluted antiserum. Similar to the conditions of the competition ELISA, each blocking antibody was used at 100 nM, and the serially diluted antisera were incubated for 1 hour. Black curves (labeled as "2nd Ab only") indicate baseline signal in the absence of both mAbs and antiserum.

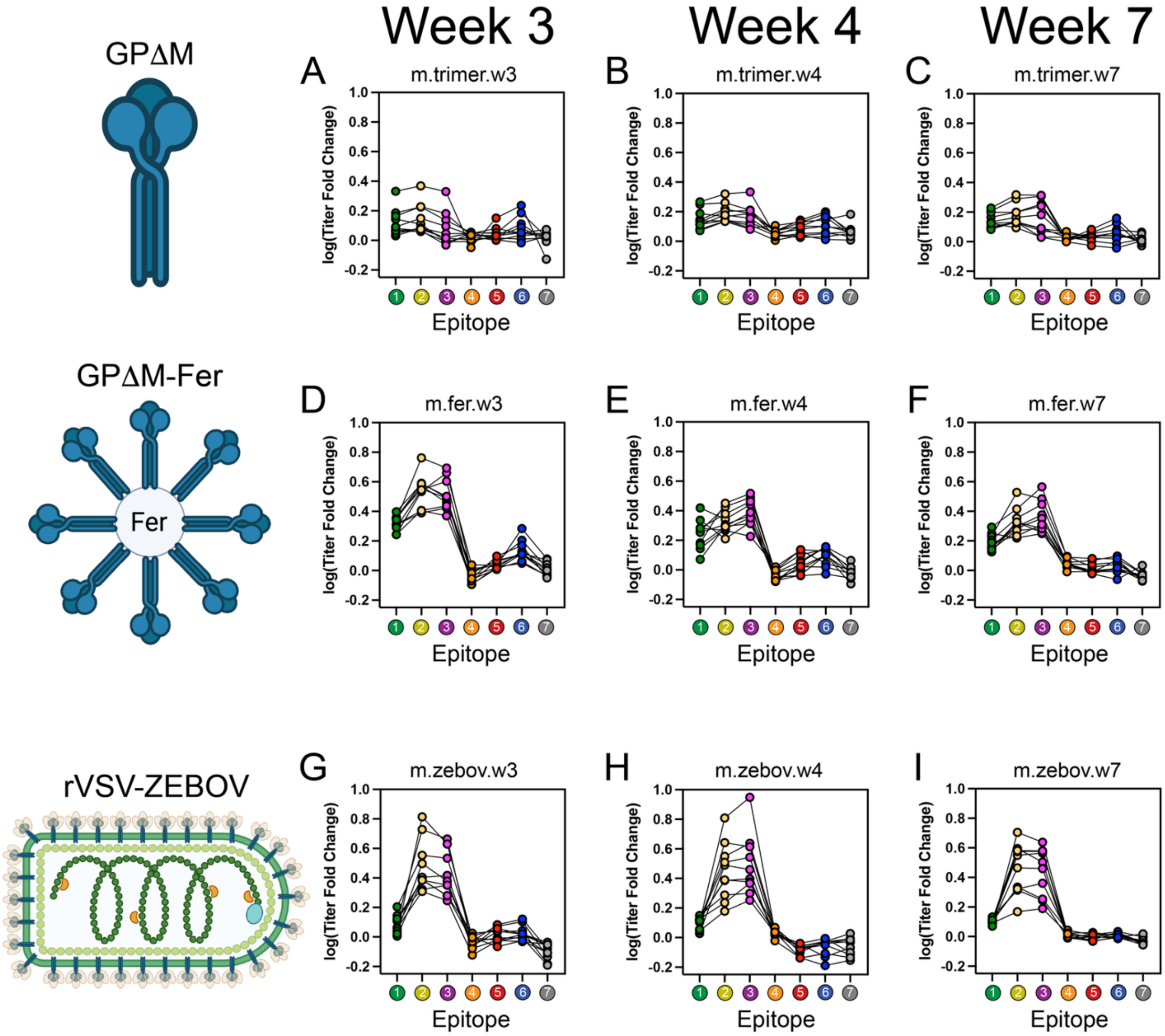

Figure S5. Individual log-transformed IgG titer fold-changes after epitope blocking in BALB/c mice across all immunization groups and timepoints. A-C, GPΔM trimer group at week 3 (**A**), week 4 (**B**), and week 7 (**C**). **D-F**, GPΔM-Fer group at week 3 (**D**), week 4 (**E**), and week 7 (**F**). **G-I**, rVSV-ZEBOV group at week 3 (**G**), week 4 (**H**), and week 7 (**I**). Titer fold-changes for each epitope were calculated as the ratio of IgG titers before and after epitope blocking.

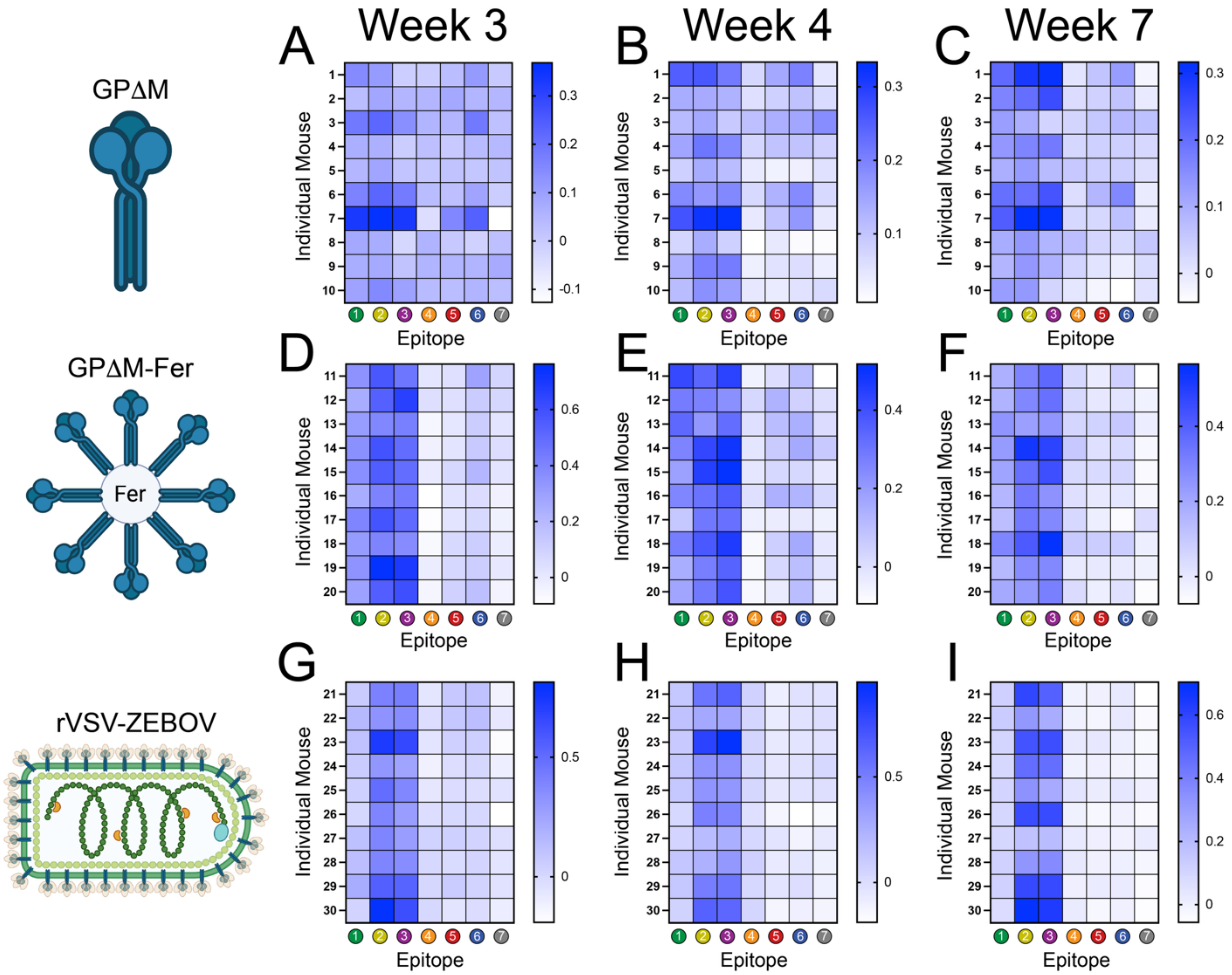

Figure S6. Heatmaps of unnormalized, log-transformed IgG titer fold-changes after epitope blocking in BALB/c mice across all immunization groups and timepoints. A-C, GPΔM trimer group at week 3 (**A**), week 4 (**B**), and week 7 (**C**). **D-F**, GPΔM-Fer group at week 3 (**D**), week 4 (**E**), and week 7 (**F**). **G-I**, rVSV-ZEBOV group at week 3 (**G**), week 4 (**H**), and week 7 (**I**). Each row represents an individual mouse, and each column corresponds to one of the seven GP epitopes. Color intensity reflects the magnitude of the log-transformed titer fold-change following mAb blocking.

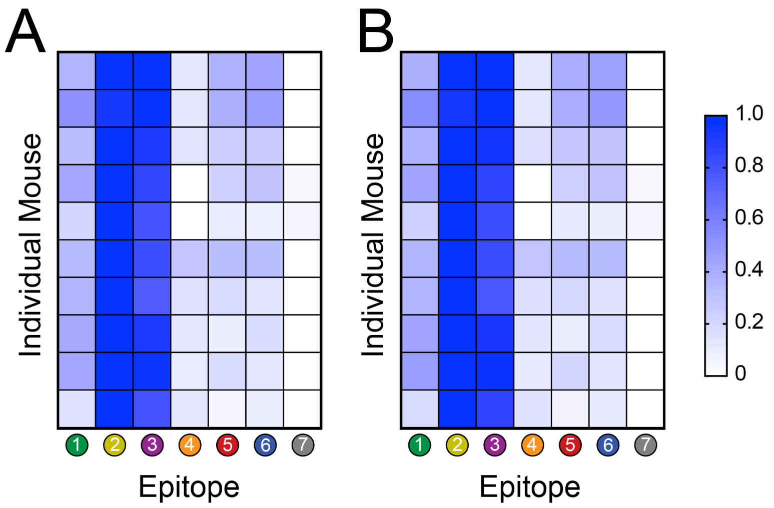

Figure S7. Comparison of epitope hierarchy determined by titer fold-change and absorbance change at the ED_{50} dilution. **A**, Heatmap of epitope hierarchy in rVSV-ZEBOV-immunized BLAB/c mice at week 3 based on log-transformed IgG titer fold-changes after epitope blocking. **B**, Heatmap of epitope hierarchy in the same animals based on changes in absorbance at 450 nm after epitope blocking at the ED_{50} dilution. In both panels, each row represents an individual mouse and each column corresponds to one of the seven GP epitopes. Color intensity reflects per-animal scaling of response values from zero to one, with blue indicating stronger responses.

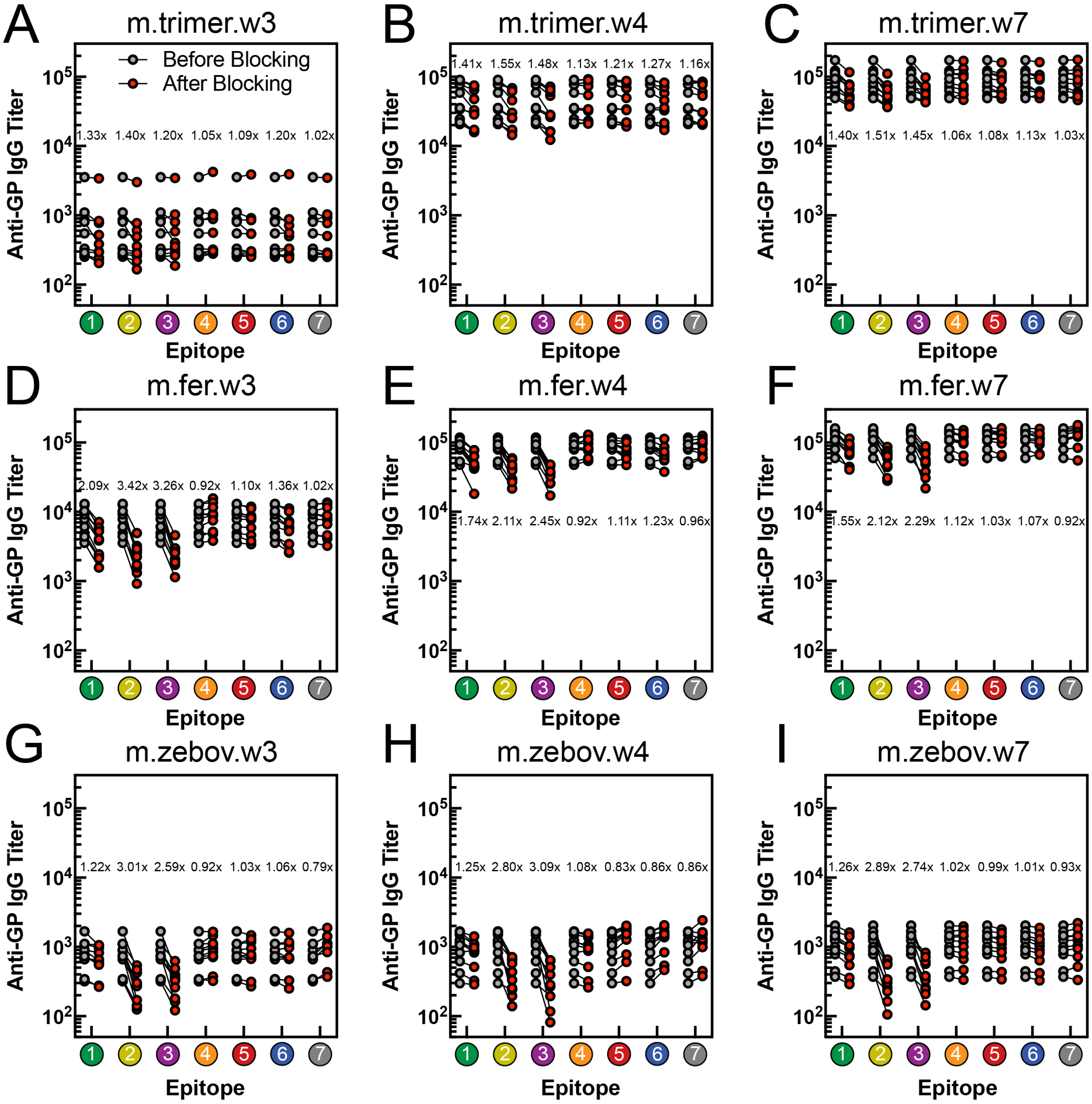

Figure S8. IgG titers of mouse antisera before and after epitope blocking across all immunization groups and timepoints. Geometric mean titer fold-changes for each epitope are shown above the corresponding column. **A**-**C**, GPΔM trimer group at week 3 (**A**), week 4 (**B**), and week 7 (**C**). **D**-**F**, GPΔM-Fer group at week 3 (**D**), week 4 (**E**), and week 7 (**F**). **G**-**I**, rVSV-ZEBOV group at week 3 (**G**), week 4 (**H**), and week 7 (**I**).

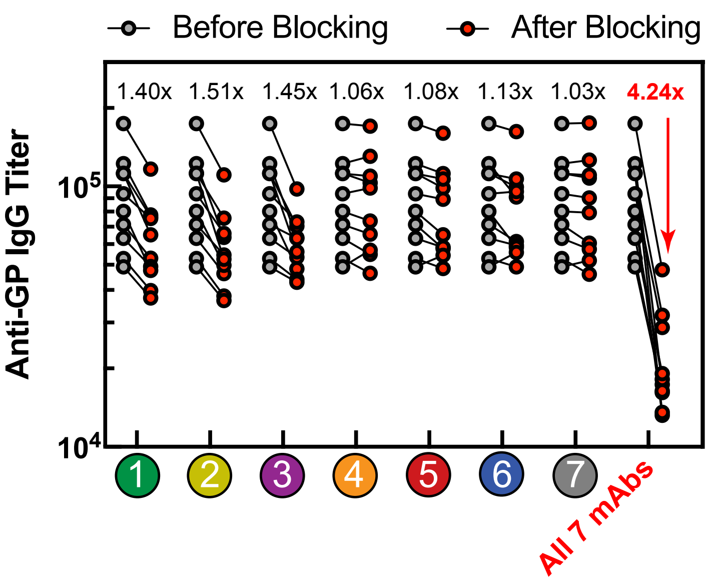

Figure S9. IgG titer fold-changes of mouse antisera following individual epitope blocking or combined blocking with all seven reference mAbs. Mouse antisera were from the GPΔM trimer group at week 7. Geometric mean titer fold-changes are shown above the corresponding column.

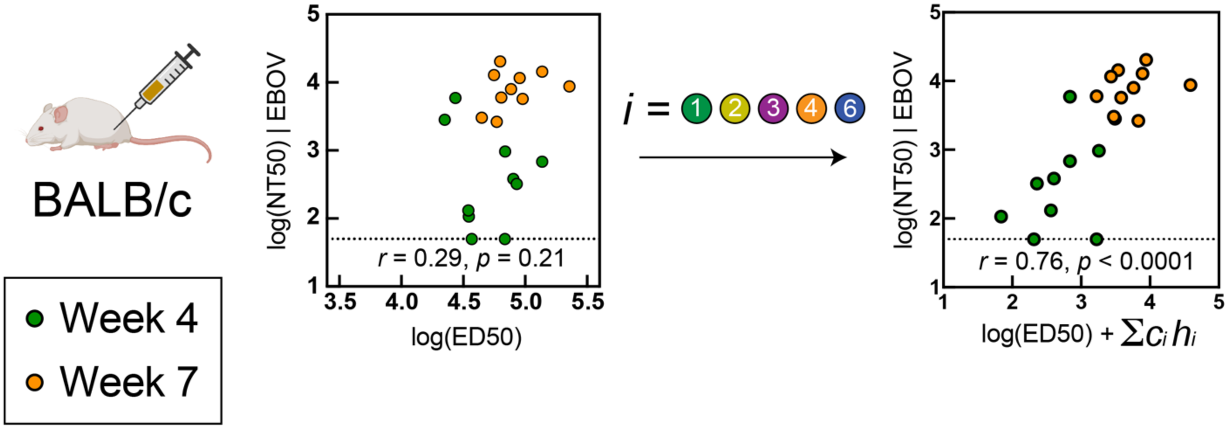

Figure S10. NT_{50}-ED_{50} correlation in the mouse GPΔM trimer group with and without incorporating epitope hierarchy. Left: log-transformed NT_{50} [log(NT_{50})] versus log-transformed ED_{50} [log(ED_{50})]. Right: log(NT_{50}) versus a linear combination of log(ED_{50}) and epitope hierarchy values (normalized log-transformed titer fold-changes) for epitopes ①②③④⑥. Epitope selection was based on the combination that yielded the highest adjusted R^2 value in linear regression analysis. In the expression $\sum c_i h_i$, c_i represents the regression coefficient for each selected epitope, and h_i represents its corresponding hierarchy value. Pearson correlation coefficients (r) and p-values are reported. Dashed lines indicate the lower limit of quantification.

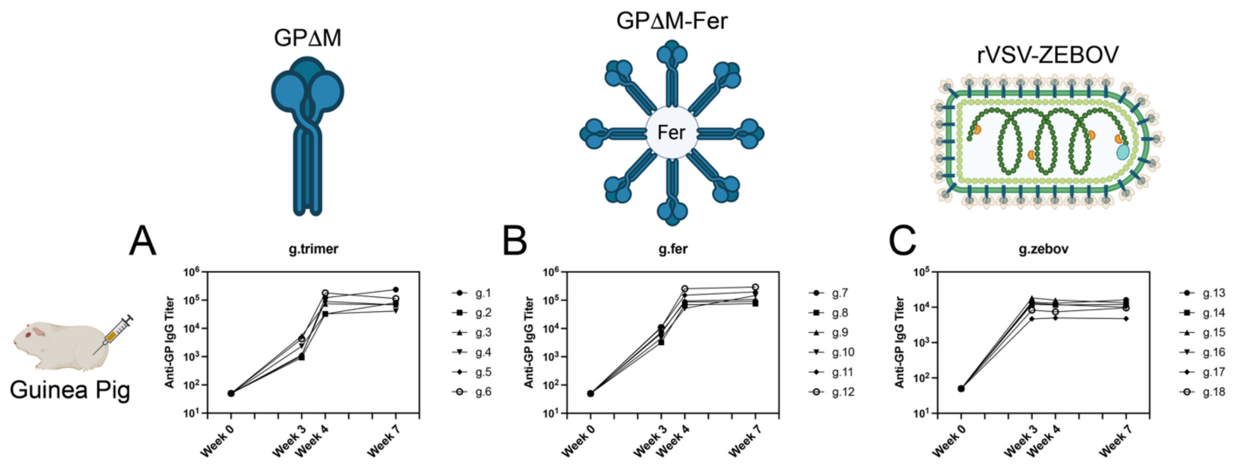

Figure S11. Individual guinea pig anti-GPΔM IgG titers across all immunization groups and timepoints. **A**, Guinea pigs immunized with GPΔM trimer. **B**, Guinea pigs immunized with GPΔM-Fer. **C**, Guinea pigs immunized with rVSV-ZEBOV. IgG titers were measured by ELISA at multiple timepoints post-immunization. GPΔM trimer and GPΔM-Fer groups received booster vaccinations at weeks 3 and 6, whereas the rVSV-ZEBOV group received a single dose without boosting.

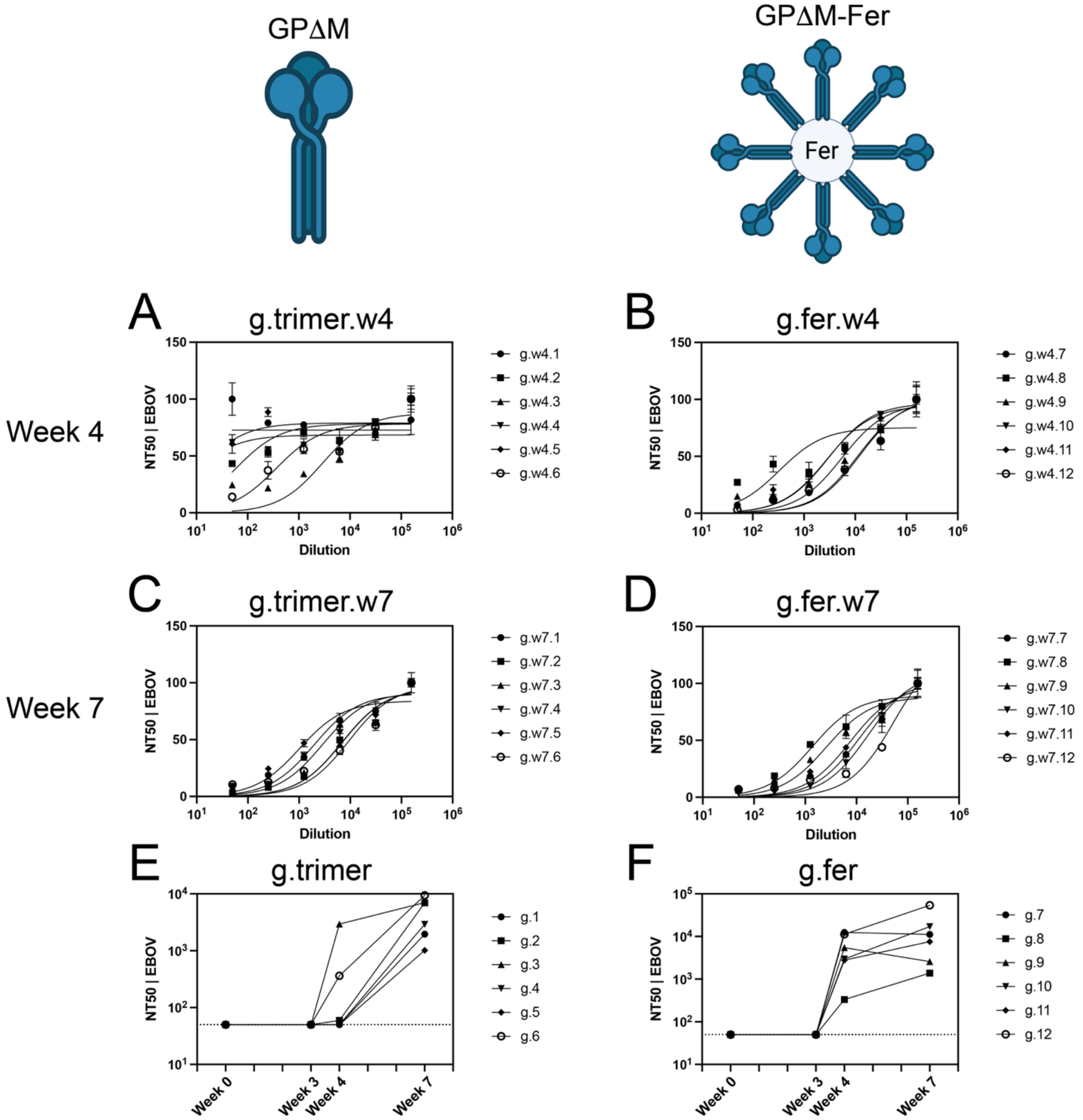

Figure S12. Pseudotyped virus neutralization titers for individual guinea pigs across all immunization groups and timepoints. A-B, Neutralization curves at week 4 for the GPΔM (**A**) and GPΔM-Fer (**B**) groups. **C-D**, Neutralization curves at week 7 for the GPΔM (**C**) and GPΔM-Fer (**D**) groups. **E-F**, Summary plots of individual neutralization titers (NT_{50}) across timepoints for the GPΔM (**E**) and GPΔM-Fer (**F**) groups. Dashed lines in **E-F** indicate the assay's lower limit of quantification.

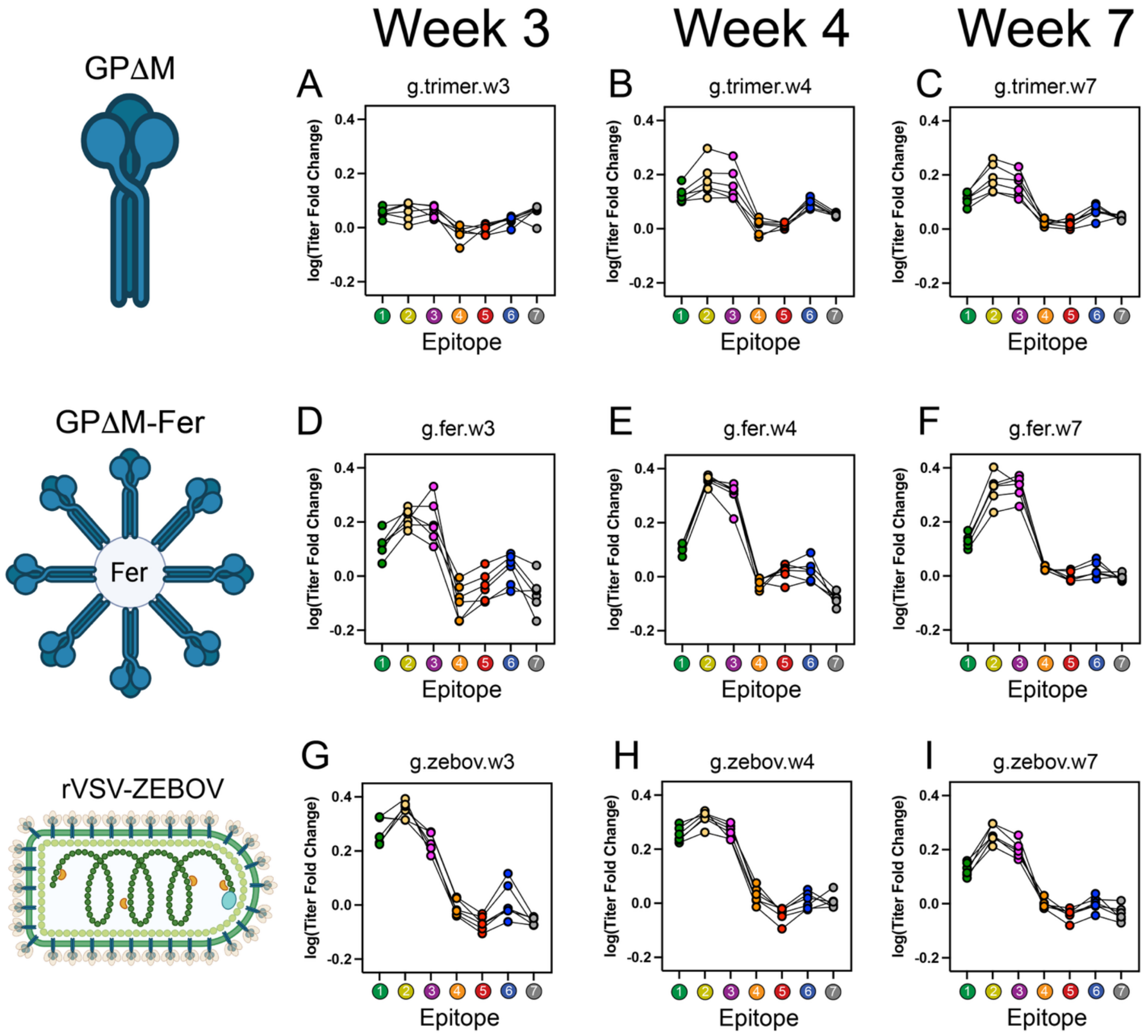

Figure S13. Individual log-transformed IgG titer fold-changes after epitope blocking in guinea pigs across all immunization groups and timepoints. A-**C**, GPΔM trimer group at week 3 (**A**), week 4 (**B**), and week 7 (**C**). **D**-**F**, GPΔM-Fer group at week 3 (**D**), week 4 (**E**), and week 7 (**F**). **G**-**I**, rVSV-ZEBOV group at week 3 (**G**), week 4 (**H**), and week 7 (**I**). Titer fold-changes for each epitope were calculated as the ratio of IgG titers before and after epitope blocking.

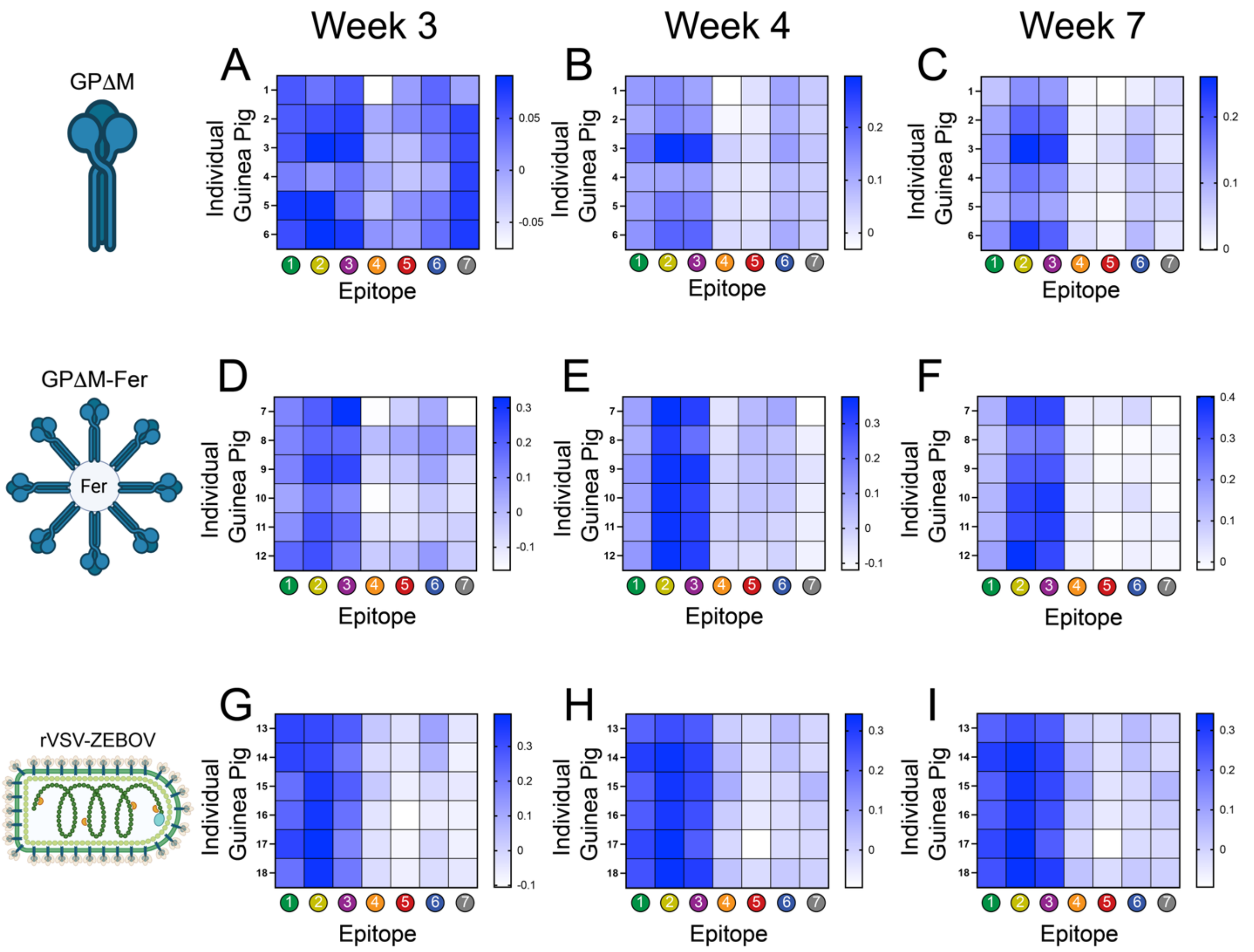

Figure S14. Heatmaps of unnormalized, log-transformed IgG titer fold-changes after epitope blocking in guinea pigs across all immunization groups and timepoints. **A**-**C**, GPΔM trimer group at week 3 (**A**), week 4 (**B**), and week 7 (**C**). **D**-**F**, GPΔM-Fer group at week 3 (**D**), week 4 (**E**), and week 7 (**F**). **G**-**I**, rVSV-ZEBOV group at week 3 (**G**), week 4 (**H**), and week 7 (**I**). Each row represents an individual guinea pig, and each column corresponds to one of the seven GP epitopes. Color intensity reflects the magnitude of the log-transformed titer fold-change following mAb blocking.

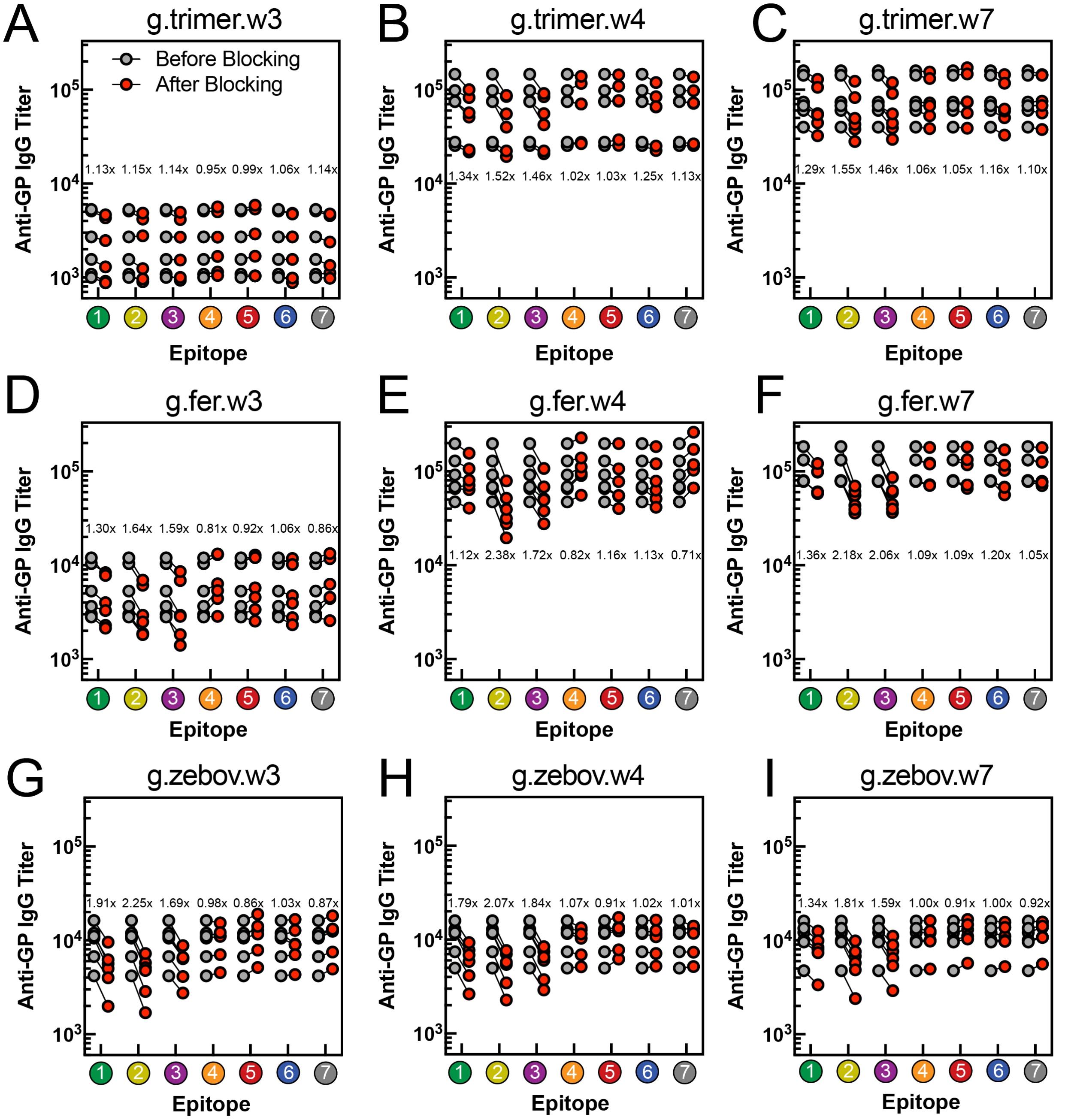

Figure S15. IgG titers of guinea pig antisera before and after epitope blocking across all immunization groups and timepoints. Geometric mean titer fold-changes for each epitope are shown above the corresponding column. **A-C**, GPΔM trimer group at week 3 (**A**), week 4 (**B**), and week 7 (**C**). **D-F**, GPΔM-Fer group at week 3 (**D**), week 4 (**E**), and week 7 (**F**). **G-I**, rVSV-ZEBOV group at week 3 (**G**), week 4 (**H**), and week 7 (**I**).

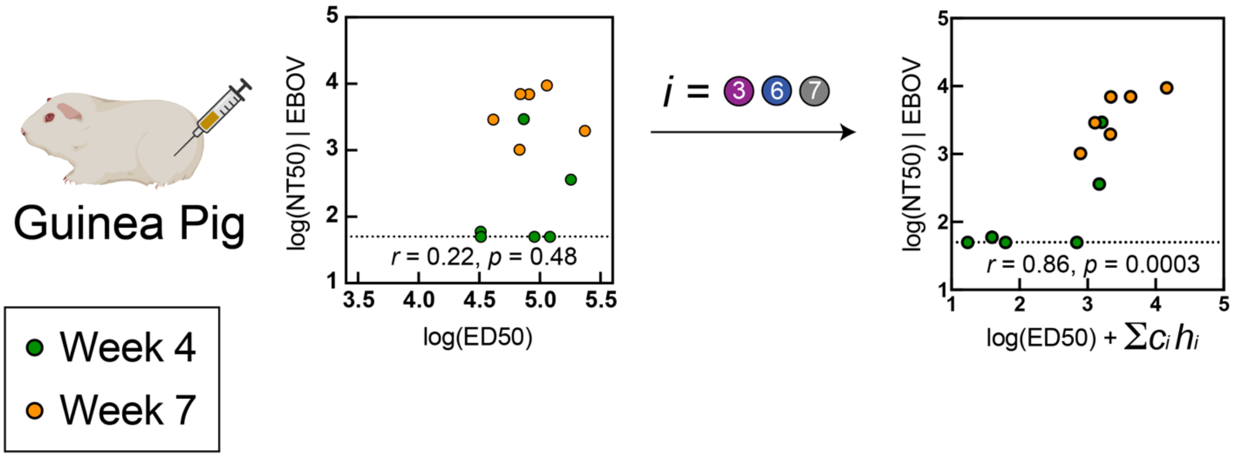

Figure S16. NT$_{50}$-ED$_{50}$ correlation in the guinea pig GPΔM trimer group with and without incorporating epitope hierarchy. Left: log(NT$_{50}$) versus log(ED$_{50}$). Right: log(NT$_{50}$) versus a linear combination of log(ED$_{50}$) and epitope hierarchy values for epitopes ③⑥⑦, selected using the same criterion as in Figure S9. In the expression $\sum c_i h_i$, c_i represents the regression coefficient for each selected epitope, and h_i represents its corresponding hierarchy value. Pearson correlation coefficients (*r*) and *p*-values are reported. Dashed lines indicate the lower limit of quantification.

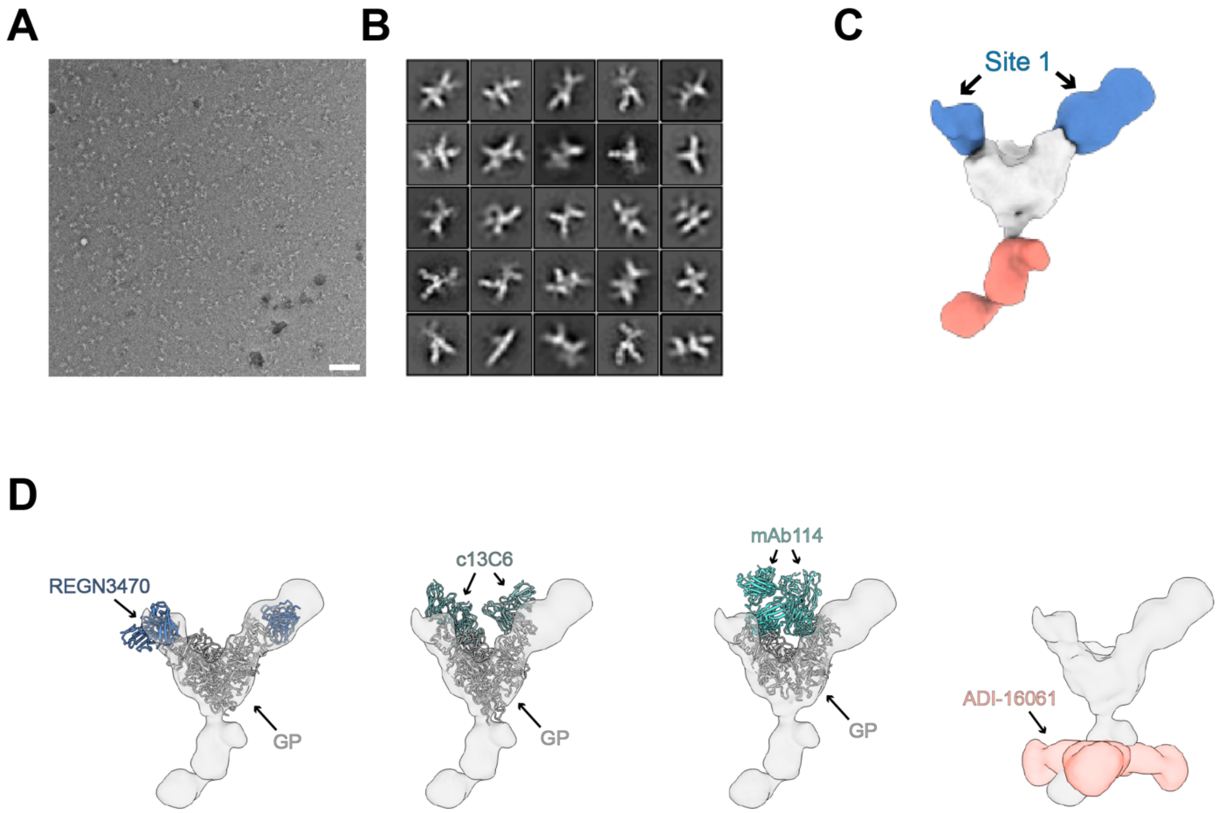

Figure S17. nsEMPEM analysis of serum antibody Fabs from guinea pig #3 immunized with GPΔM. **A**, Representative micrograph of GPΔM complexed with serum antibody Fabs. Scalebar (white) represents 100 nm. **B**, Representative 2D classes of GPΔM complexed with serum antibody Fabs. **C**, Composite maps of GPΔM complexed with serum antibody Fabs. **D**, Published Fab-GP structures docked into the composite map from panel C. Coordinates used are from the following PDBs: REGN3470-GP (PDB 7TN9), c13C6-GP (PDB 5KEL), and mAb114-GP (PDB 5FHC). Map of ADI-16061 is from EMD-8698.

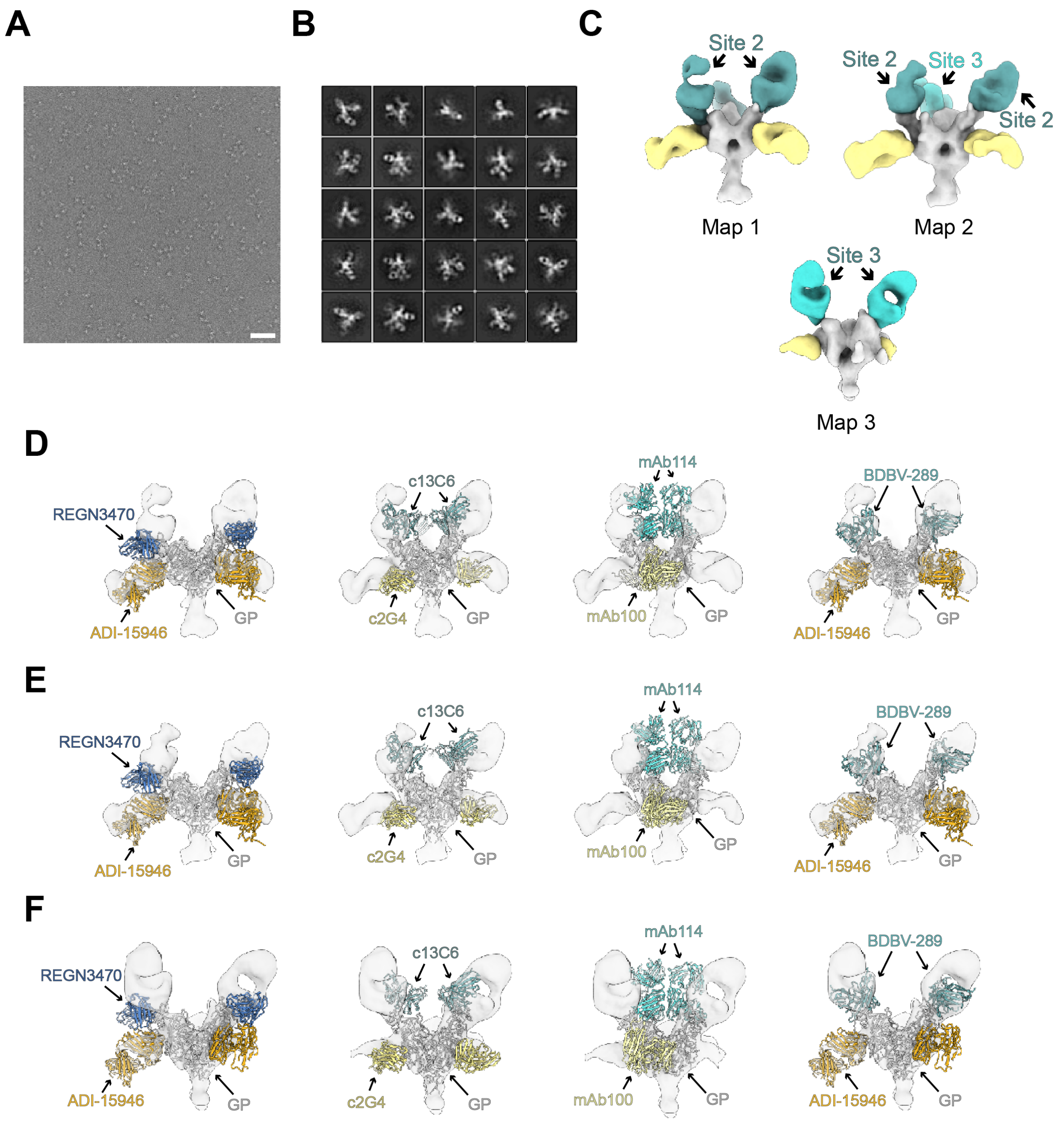

Figure S18. nsEMPEM analysis of serum antibody Fabs from guinea pig #10 immunized with GPΔM-Fer. A, Representative micrograph of GPΔM complexed with serum antibody Fabs. Scalebar (white) represents 100 nm. **B**, Representative 2D classes of GPΔM complexed with serum antibody Fabs. **C**, Composite maps of GPΔM complexed with serum antibody Fabs. **D** – **F**, Published Fab-GP structures docked into Map 1 (**D**), Map 2 (**E**), and Map 3 (**F**). Coordinates used are from the following PDBs: REGN3470-GP (PDB 7TN9), ADI-15949-GP (PDB 6MAM), c13C6 and c2G4 bound to GP (PDB 5KEL), mAb114 and mAb100 bound to GP (PDB 5FHC), and BDBV-289-GP (PDB 7KEJ).

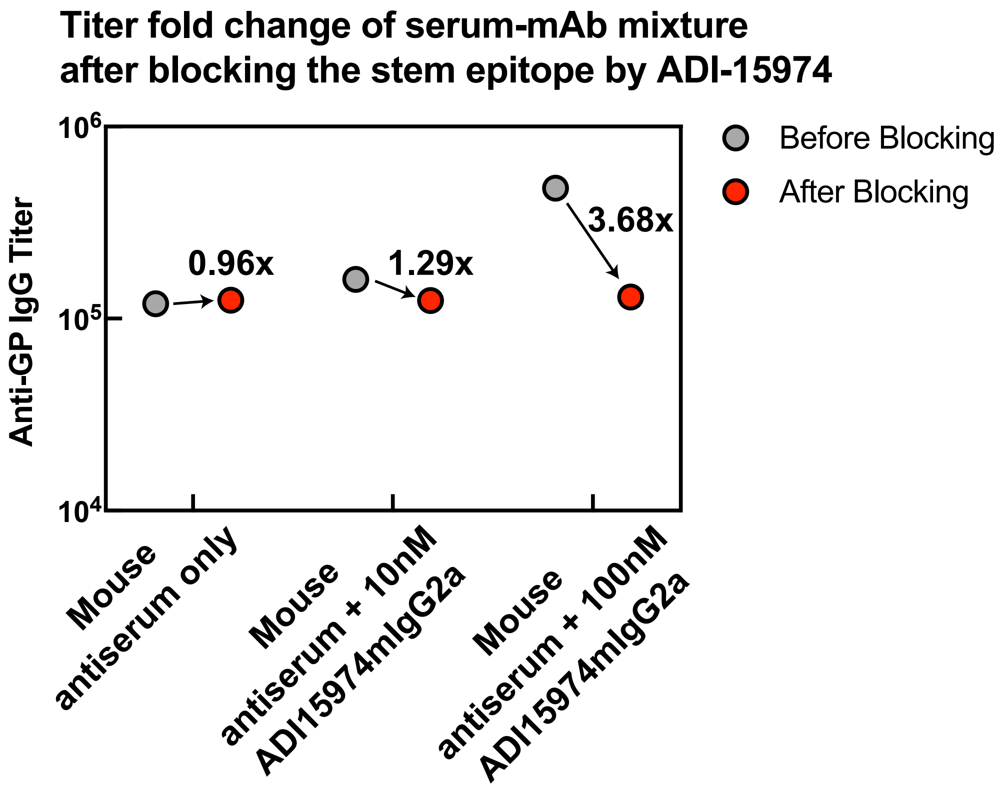

Figure S19. Titer fold change of mouse antisera after epitope blocking by ADI-15974 when mixed with varying concentrations of the mouse IgG version of ADI-15974 (ADI15974mIgG2a). Adding ADI15974mIgG2a increased the pre-blocking binding titer while yielding a similar post-blocking titer, resulting in larger titer fold-changes at higher ADI15974mIgG2a concentrations.

Supplementary Tables

Table S1. Neutralization potencies of the antibodies used for epitope mapping.

**Antibody**	**Reported IC_{50} (nM)**
REGN3470[5]	0.27
c13C6[6,7]	Non-neutralizing
mAb114[6]	0.6
mAb100[6]	0.4
c2G4[8]	0.93
ADI-15946[9]	0.81
ADI-15974[10]	< 0.33

Table S2. Pearson correlation between log-transformed NT_{50} and ED_{50} values in BALB/c mice using data from weeks 4 and 7, or from weeks 3, 4, and 7.

	GPΔM trimer		GPΔM-Fer	
	r	*p*	*r*	*p*
Week 4 + Week 7	0.29	0.21	0.71	0.0004
Week 3 + Week 4 + Week 7	n/a	n/a	0.96	< 0.0001

REFERENCES

(1) Glasgow, A.; Glasgow, J.; Limonta, D.; Solomon, P.; Lui, I.; Zhang, Y.; Nix, M. A.; Rettko, N. J.; Zha, S.; Yamin, R. Engineered ACE2 Receptor Traps Potently Neutralize SARS-CoV-2. *Proc. Natl. Acad. Sci. U.S.A.* **2020**, *117* (45), 28046-28055.

(2) Xu, D.; Powell, A. E.; Utz, A.; Sanyal, M.; Do, J.; Patten, J.; Moliva, J. I.; Sullivan, N. J.; Davey, R. A.; Kim, P. S. Design of Universal Ebola Virus Vaccine Candidates via Immunofocusing. *Proc. Natl. Acad. Sci. U.S.A.* **2024**, *121* (7), e2316960121.

(3) Bruun, T. U.; Do, J.; Weidenbacher, P. A.-B.; Utz, A.; Kim, P. S. Engineering a SARS-CoV-2 Vaccine Targeting the Receptor-Binding Domain Cryptic-Face via Immunofocusing. *ACS Cent. Sci.* **2024**, *10* (10), 1871-1884.

(4) Turner, H. L.; Jackson, A. M.; Richey, S. T.; Sewall, L. M.; Antanasijevic, A.; Hangartner, L.; Ward, A. B. Protocol for Analyzing Antibody Responses to Glycoprotein Antigens Using Electron-Microscopy-Based Polyclonal Epitope Mapping. *STAR protocols* **2023**, *4* (3), 102476.

(5) Pascal, K. E.; Dudgeon, D.; Trefry, J. C.; Anantpadma, M.; Sakurai, Y.; Murin, C. D.; Turner, H. L.; Fairhurst, J.; Torres, M.; Rafique, A. Development of Clinical-Stage Human Monoclonal Antibodies That Treat Advanced Ebola Virus Disease in Nonhuman Primates. *J. Infect. Dis.* **2018**, *218*, S612-S626.

(6) Corti, D.; Misasi, J.; Mulangu, S.; Stanley, D. A.; Kanekiyo, M.; Wollen, S.; Ploquin, A.; Doria-Rose, N. A.; Staupe, R. P.; Bailey, M.; et al. Protective Monotherapy against Lethal Ebola Virus Infection by a Potently Neutralizing Antibody. *Science* **2016**, *351* (6279), 1339-1342.

(7) Misasi, J.; Sullivan, N. J. Immunotherapeutic Strategies to Target Vulnerabilities in the Ebolavirus Glycoprotein. *Immunity* **2021**, *54* (3), 412-436.

(8) Audet, J.; Wong, G.; Wang, H.; Lu, G.; Gao, G. F.; Kobinger, G.; Qiu, X. Molecular Characterization of the Monoclonal Antibodies Composing Zmab: A Protective Cocktail against Ebola Virus. *Sci. Rep.* **2014**, *4* (1), 6881.

(9) West, B. R.; Wec, A. Z.; Moyer, C. L.; Fusco, M. L.; Ilinykh, P. A.; Huang, K.; Wirchnianski, A. S.; James, R. M.; Herbert, A. S.; Hui, S. Structural Basis of Broad Ebolavirus Neutralization by a Human Survivor Antibody. *Nat. Struct. Mol. Biol.* **2019**, *26* (3), 204-212.

(10) Bornholdt, Z. A.; Turner, H. L.; Murin, C. D.; Li, W.; Sok, D.; Souders, C. A.; Piper, A. E.; Goff, A.; Shamblin, J. D.; Wollen, S. E. Isolation of Potent Neutralizing Antibodies from a Survivor of the 2014 Ebola Virus Outbreak. *Science* **2016**, *351* (6277), 1078-1083.
